# ﻿Review of the *Idaeaproximaria* complex (Lepidoptera, Geometridae, Sterrhinae) with descriptions of four new species

**DOI:** 10.3897/zookeys.1169.106815

**Published:** 2023-07-18

**Authors:** Rui Cheng, Da-Yong Xue, Nan Jiang, Hong-Xiang Han

**Affiliations:** 1 Key Laboratory of Zoological Systematics and Evolution, Institute of Zoology, Chinese Academy of Sciences, Beijing 100101, China Institute of Zoology, Chinese Academy of Sciences Beijing China

**Keywords:** China, endemic, tarsus, variation

## Abstract

The *Idaeaproximaria* complex is reviewed and four new species are described from China: *Idaearectangularis* Cheng & Han, **sp. nov.** from Guangxi and Fujian provinces, *Idaearectispina* Cheng & Han, **sp. nov.** from Hunan province, *Idaeasetosa* Xue & Han, **sp. nov.** from Hainan province, and *Idaealinearis* Xue & Han, **sp. nov.** from Hubei and Shaanxi provinces. Illustrations of adults and genitalia of the new species and known species are presented, and the variations in the form of the aedeagus of *Idaeaproximaria* are discussed.

## ﻿Introduction

*Idaea* Treitschke is the third most species-rich genus in the Geometridae, including 1080 valid species (Rajaei et al. 2022) with a worldwide distribution. Sterneck (1940) subdivided the genus (as *Sterrha*) into 34 species groups based on characters of the aedeagus. Hausmann (2004) largely followed his partition and divided 107 European species and many species distributed in adjacent countries into 26 species groups. However, *Idaeaproximaria* (Leech, 1897) was not included in these two important publications, because it is a species endemic to China. *Idaeaproximaria* was originally described by Leech (1897) in the genus *Chrysocraspeda*, based on two male specimens from Moupin, Sichuan, and Leech stated that it was allied to *Hyriamarginata* Swinhoe. Prout (1913) placed this species in Ptychopoda (Section B. *Ptychopoda*) on the basis of the lack of terminal spurs on the male hind tibia, and provided a description and comparison with *Chrysocraspedamarginata*. Later, Prout (1938) placed it in the genus Sterrha (Section B. *Ptychopoda*), and compared it to *Sterrhacraspedota* Prout (replacement name for *Hyriamarginata* Swinhoe). In the geometrid catalogue of Scoble (1999), both *proximaria* and *craspedota* are listed in the genus *Idaea*. The main differences between *I.proximaria* and *I.craspedota* are that the forewing areole is present in *I.proximaria* but absent in *I.craspedota*, and while the first male hind tarsus is slightly dilated in *I.craspedota*, in *I.proximaria* the modification of the male hind tarsus is very distinctive, with the first segment of hind tarsus broad and flat, shaped like a bird’s wing, and a long hair-pencil is present from the base of the tibia to the tip of the tarsus.

Further study of the specimens of *Idaeaproximaria* from IZCAS shows that four new species need to be described. The purposes of this paper are to provide a survey of the *Idaeaproximaria* complex, to describe four new species, and to provide illustrations of the external features and genitalia of all the species.

## ﻿Materials and methods

Specimens used are from the Institute of Zoology, Chinese Academy of Sciences, Beijing, China (**IZCAS**). Terminology for the genitalia is based on Pierce (1914, reprinted 1976), Klots (1970), and Nichols (1989). Moths were photographed with a digital camera (Canon Pc1057). Composite images were generated using Auto-Montage software v. 5.03.0061 (Synoptics Ltd). The sharpness-contrast of the photos was enhanced and the plates compiled using Adobe Photoshop (CS 5.1).

Four specimens of *I.proximaria* were used for sequencing the DNA barcoding region of the mitochondrial COI gene, and DNA barcodes (658 bp) of these four specimens were obtained. Three sequences of the related species *I.craspedota* were downloaded from BOLD. Protocols of DNA extraction and sequencing followed Ban et al. (2018). Details of studied specimens, including GenBank accession numbers are summarised in Table 1. A neighbour-joining (NJ) tree (Saitou and Nei 1987) was constructed based on the Kimura two-parameter (K2P) method (Kimura 1980) using MEGA 6.0. In addition, to explore the taxonomic position of *proximaria* complex, some other *Idaea* sequences were downloaded and constructed the NJ tree.

**Table 1.** Details of specimens used in molecular analysis of the DNA barcode region.

Sample ID	Species	Date collected	Locality	Collectors	GenBank accession number
LEP M 31611	*I.proximaria*	16–19.vii.2017	Jinsixia, Shaanxi	Cui L	OR094697
LEP M 31612	*I.proximaria*	16–19.vii.2017	Jinsixia, Shaanxi	Cui L	OR094698
LEP M 31619	*I.proximaria*	16–19.vii.2017	Jinsixia, Shaanxi	Cui L	OR094699
LEP M 31623	*I.proximaria*	16–19.vii.2017	Jinsixia, Shaanxi	Cui L	OR094700
RMNH.INS.13917	*I.craspedota*	28.xi.2005	Indonesia	EJ van Nieukerken, E Gassó	HM387102
RMNH.INS.13918	*I.craspedota*	29.xi.2005	Indonesia	EJ van Nieukerken	GU662733
RMNH.INS.14291	*I.craspedota*	14.xi.2005	Indonesia	R de Jong	GU662632

## ﻿Systematics

### ﻿*Idaeaproximaria* complex

**Figures 1–15.**
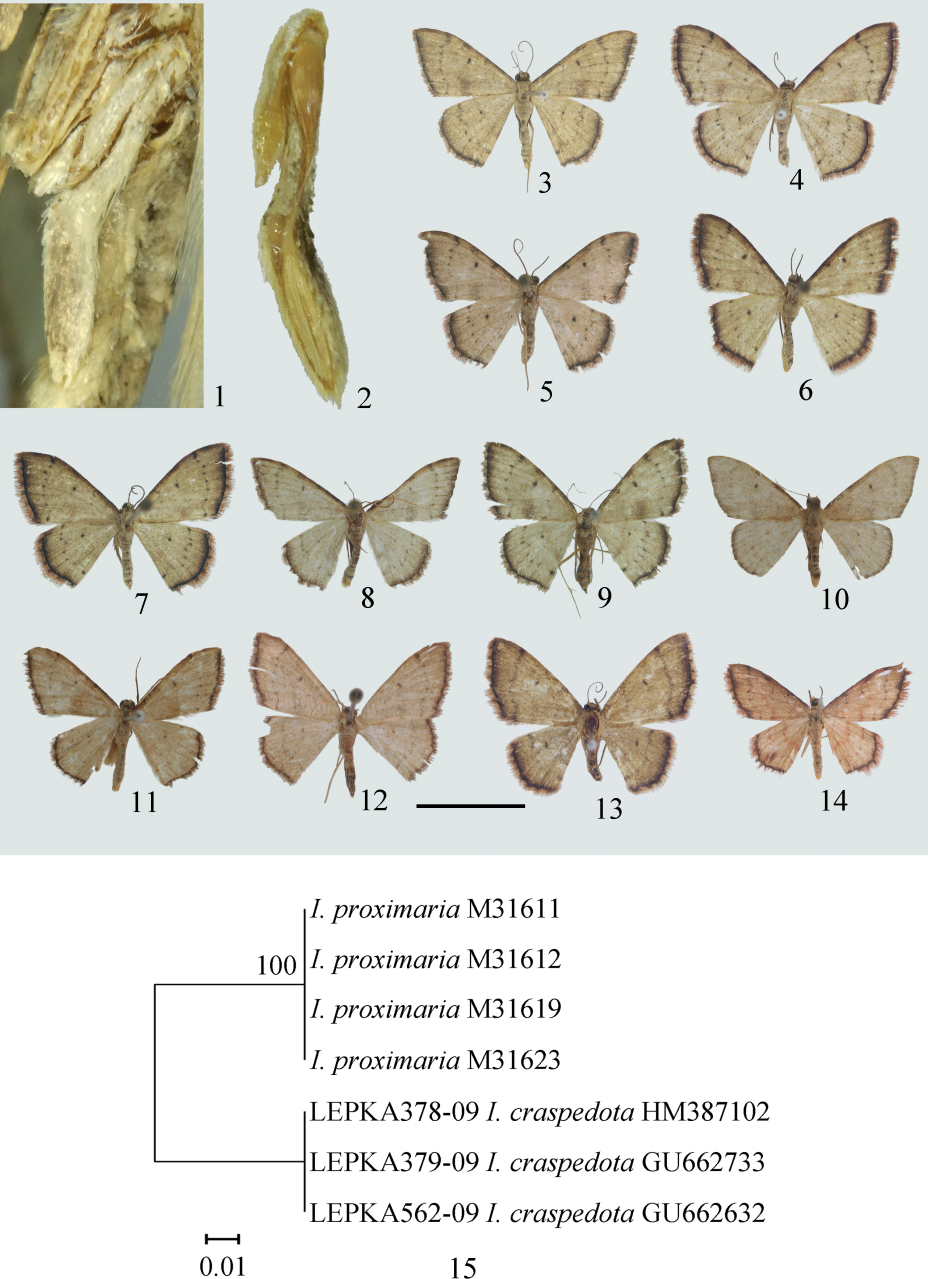
(**1–2**) Male hind tibia and tarsus of *Idaeaproximaria***1** lateral view **2** medial view. (**3–14**) Adults of *Idaeaproximaria* complex **3–7***I.proximaria***3** male, Jiguanshan, Sichuan **4** female, Baoxing, Sichuan **5–7** Jinsixia, Shaanxi **5–6** male **7** female **8–9***I.rectangularis* sp. nov. **8** holotype, male, IZCAS**9** paratype, female, IZCAS**10***I.rectispina* sp. nov., holotype, male, IZCAS**11–12***I.setosa* sp. nov. **11** holotype, male, IZCAS**12** paratype, female, IZCAS**13***I.linearis* sp. nov., holotype, female, IZCAS**14***I.craspedota*. **15** Neighbour-Joining (NJ) tree of selected *proximaria* complex based on the Kimura two-parameter model. Scale bar: 1 cm (**3–14**).

No treatment of the species group of *I.proximaria* was found in the literature. It is not easy to place it into the known species groups, and it can be regarded as a separate species group, named the *proximaria* complex in the present work. The species in the *proximaria* complex share the following characters: antennae ciliate in male and filiform in female; frons flat, black; male hind tibia without spurs, first segment of hind tarsus very broad and flat, modified into the shape of a bird’s wing (Figs 1, 2); female hind tibia with a pair of terminal spurs; adults with pale brown ground colour, small discal dot present on both wings, postmedial line usually appearing as small dots on veins, terminal margin a narrow dark purplish brown band; male genitalia with broad uncus, aedeagus with various numbers of cornuti; female genitalia with ductus bursae often broad, bearing an elongate appendix bursae, corpus bursae small or large, spinulose.

Based on the total available *Idaea* sequences, a NJ tree with weak support value (Suppl. material 1) still failed to solve the phylogenetic relationships of the *proximaria* complex and its taxonomic position within *Idaea* remains unclear. The modification of the first male hind tarsus is reminiscent of those of *I.filicata* (Hübner), *I.troglodytaria* (Heydenreich), and *I.efflorata* Zeller, with text figures provided in Hausmann (2004: figs 18, 19, 21), which are placed in *rusticata* species group. This modification may indicate a close relationship between the two groups. However, the tarsus is vestigial and only the hind tibia is terminally dilated into a spoon-shape in *I.filicata* and *I.troglodytaria* (Hausmann, 2004: 91, 92). The hair-pencil of the *proximaria* complex is very long and extends to the tip of the tarsus, but is shorter and covering only 2/3 of the tarsus in *I.efflorata* (Hausmann, 2004: 96). In addition, the male hind tibia of some other species including *I.rusticata* (Denis & Schiffermüller) in the *rusticata* species group bear spurs, which are absent in species assigned to the *proximaria* complex.

#### ﻿ Idaea proximaria

Taxon classificationAnimaliaLepidopteraGeometridae

(Leech, 1897)

6E0AE6BB-85D1-5C77-B801-E1A2A59ED86E

Figs 3–7 , 16–19 , 24–26 , 30

Chrysocraspeda proximaria Leech, 1897: 106. Syntypes 2♂, China (western): Moupin (NHMUK).
Ptychopoda proximaria : Prout 1913: 101.
Sterrha proximaria : Prout 1934: 414.
Idaea proximaria : Scoble 1999: 504.

##### Material examined.

**China: Shaanxi** (IZCAS): 1♂, Ningshan, Huoditang, 1497 m, 29–31.VII.2018, leg. Zhang Xinyi; 1♂1♀, Taibai, Huangbaiyuan, 1279 m, 15–17.VII.2018, leg. Zhang Xinyi; 2♂, Foping, Yueba, 1052 m, 1–3.VIII.2018, leg. Zhang Xinyi; 1♀, Ningshan, Guanghuojie, Baohuzhan, 1189 m, 26–28.VII.2014, leg. Liu Shuxian, slide no. Geom-5167; 11♂1♀, Ningshan, Guanghuojie, 1101 m, 27–28.VII.2018, leg. Zhang Xinyi, slide no. Geom-5928 (♂); 13♂3♀, Shangnan, Jinsixia, 766–777 m, 23–25.VII.2013, 16–19.VII.2017, leg. Cui Le, slide no. Geom-5136 (♂), 5137 (♂), 5138 (♀), 5163 (♂), 5164 (♂), 5165 (♂), 5166 (♂), 5168 (♀), 5184 (♂), 5185 (♂). **Hubei** (IZCAS): 13♂2♀, Shennongjia, Chaoshuihe, 860 m, 21–23.VI.2019, leg. Cheng Rui. **Sichuan** (IZCAS): 1♂4♀, Emeishan, Qingyinge, 800–1000 m, 19.VI.–15.VII.1957, leg. Zhu Fuxing et al., slide no. Geom-5162 (♀); 5♂, Mianzhu, Jiulongshan, Shizipo, 810 m, 29–31.VII.2016, leg. Cui Le, slide no. Geom-4134 (♂), 5158 (♂), 5186 (♂); 8♂5♀, Baoxing, Fengtongzhai, Dashuigou, 1590 m, 1–5.VIII.2016, leg. Cui Le, slide no. Geom-4135 (♀), 5180 (♂); 3♂1♀, Chongzhou, Jiguanshan, Anzihe, Shaoyaogou, 1556 m, 11–16.VII.2016, leg. Cui Le, slide no. Geom-4141 (♂); 11♂12♀, Mianzhu, Dajianping, 877 m, 17–18.VII.2019, leg. Zhang Xinyi; 6♂7♀, Wenchuan, Huangjiacun, 1033 m, 13–14.VII.2019, leg. Zhang Xinyi; 13♂11♀, Pengzhou, Dingjiawanping, 1088 m, 15–16.VII.2019, leg. Zhang Xinyi; 6♂7♀, Tianquan, Lianglukou, 1405 m, 1–2.VII.2019, leg. Zhang Xinyi.

##### Diagnosis.

As Prout (1913, 1938) stated, *I.proximaria* is similar to *I.craspedota* (Fig. 14) in the wing pattern, but the latter lacks the areole on the forewing. The genitalia of the two species are quite different: the uncus is truncate with tiny protuberances in *I.proximaria*, but tapering and pointed in *I.craspedota* (Fig. 23); the gnathos is developed in *I.proximaria*, but only bears a blunt median process in *I.craspedota*; the valva has different decorations in *I.proximaria*, but is very narrow and simple in *I.craspedota*; the anellus is very large in *I.proximaria* but reduced in *I.craspedota*. In the female genitalia, the ductus bursae of *I.proximaria* is very broad but quite narrow in *I.craspedota* (Fig. 34).

##### Redescription.

***Head*.** Antennae ciliate in male, filiform with sparse cilia in female, cilia shorter in female. Frons dark, with sparsely scattered brown scales, not protruding. Labial palpus dark brown on dorsal side and paler on ventral side, third segment ~ 1/2 length of second segment, extending beyond frons. Vertex pale brown.

***Thorax*.** Tegula pale brown. Hind leg in male modified; hind tibia dilated; first segment of tarsus very broad and flat, shaped like a bird’s wing, covered with darkish scales, the inner side bearing a bunch of long hair-pencils from base of tibia to tip of tarsus, other tarsomeres vestigial. Hind tibia in female normal, with one pair of terminal spurs. Forewing length: male 9–11 mm, female 9–12 mm. Wings pale brown, diffused with dark scales. Forewing with costa straight, distal one-third convex; apex protruding; outer margin slightly convex. Forewing with costa dark purplish brown; antemedial line vague, discernible by a small patch on costal margin; postmedial line sinuous, appearing as black dots on veins, vague between veins; terminal margin a narrow dark purplish brown band; an indistinct darkish patch present at middle of hind margin. Hind wing with outer margin slightly protruding on vein M_3_; postmedial line and terminal margin same as on forewing. Both wings with black discal spot. Fringes reddish brown, decorated with dark scales on vein ends. Underside: paler than upperside; forewing with basal part diffused with greyish scales; postmedial line more continuous than on upperside; terminal band much narrower.

***Abdomen*.** Dorsal side pale brown, decorated with dark brown scales, dense on intersegment; ventral side pale brown.

***Male genitalia*.** Uncus spatulate, with two tiny lateral and ventral protuberances at tip. Gnathos developed, tongue-like. Valva long and slender, apex rounded and setose; dorsal margin almost straight to slightly convex, with a subapical mound-like protrusion; ventral margin with a blunt central protrusion. Anellus a very large sclerite, posterior margin smooth and strongly protruding, lateral margin with lower half spinulose. Saccus blunt. Aedeagus short, terminal two-thirds sclerotised and stout, cornutus with one or two spines.

***Female genitalia*.** Ovipositor lobes with small ventral protrusion. Apophyses posteriores ~ 2× length of apophyses anteriores. Region around ostium strongly sclerotised, lamella postvaginalis and antevaginalis irregularly shaped. Ductus bursae very long and broad, wrinkled, with a large scobinate area, anteriorly membranous, the middle part decorated with long spines, posteriorly, with a well sclerotised pouch-like process; an elongate diverticulum present, diverging posteriorly. Corpus bursae small, with a rounded spinose patch.

##### Distribution.

China (Shaanxi, Hubei, Sichuan).

##### Remarks.

It has not been possible to examine the genitalia of the syntypes of *I.proximaria* since neither of them has been dissected. However, since the syntypes and the complete series in the NHMUK (the Natural History Museum, London, United Kingdom) are from Sichuan and we have only seen one species in the complex from Sichuan province, it is reasonable to assume that this is the species described by Leech.

**Figures 16–27.**
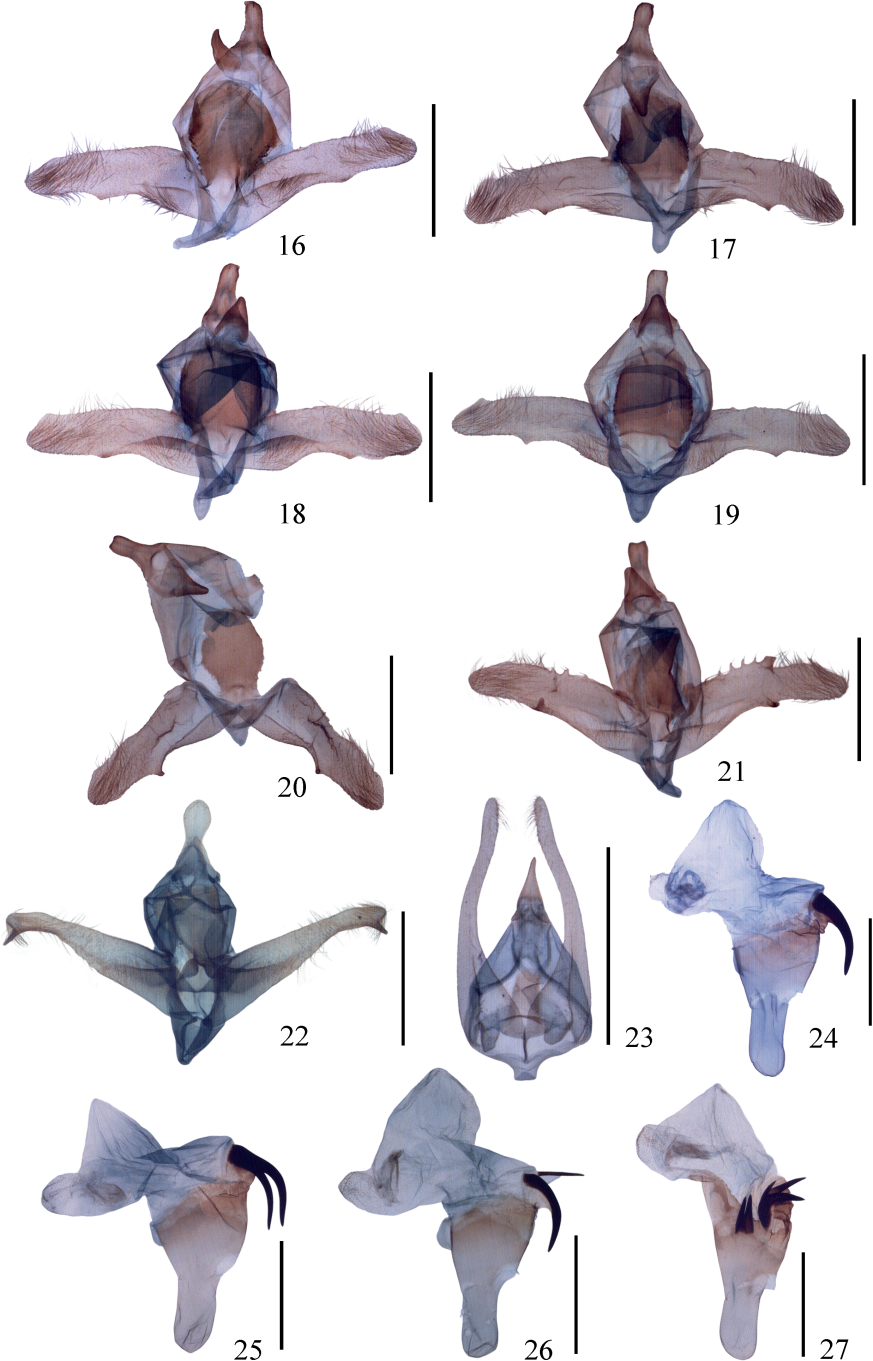
(**16–23**) Male genitalia of *Idaeaproximaria* complex **16–19***I.proximaria***16–17** Shizipo, Sichuan **18–19** Jinsixia, Shaanxi **20***I.rectangularis* sp. nov., holotype **21***I.rectispina* sp. nov., holotype **22***I.setosa* sp. nov., holotype **23***I.craspedota*. (**24–27**) Aedeagus of *I.proximaria* complex **24–26***I.proximaria***24** Dashuigou, Sichuan **25** Shizipo, Sichuan **26** Jinsixia, Shaanxi **27***I.rectangularis* sp. nov., holotype. Scale bars: 1 mm.

In *I.proximaria*, the aedeagus has a varying number of cornuti on the vesica. For example, some only have one large bent spine (Fig. 24); some have two spines of equal length (Fig. 25), or of different sizes (Fig. 26). The conditions of one spine and two equally sized spines occur among the specimens collected from Shizipo, Sichuan province, and that of two spines of different sizes occur among specimens collected from Jinsixia, Shaanxi province. Other differences could not be found in the armament of the male genitalia (Figs 16–19) or in the female genitalia and wings (Figs 3–7). To determine whether these differences are of intraspecific variations or different species, four fresh specimens from Jinsixia, Shaanxi province were used to obtain barcoding sequences; of these specimens, LEP M 31611 and 31619 bear two equal-sized spines, LEP M 31612 bears two differently sized spines, and LEP M 31623 is a female. The result (Fig. 15) shows that no genetic distance can be found between the four specimens; thus, these specimens are considered to belong to the same species. The genetic distance between *I.proximaria* and *I.craspedota* is 8.36% (Fig. 15).

Most public *Idaea* sequences on BOLD were downloaded, including 32 sequences from China, and a NJ tree was constructed (Suppl. material 1). In the NJ tree, *I.proximaria* is clustered with two unnamed specimens with very low support values, which means this relationship is unreliable. Through calculating pairwise distances, *I.politaria* (Hübner) of *inquinata* species group was found as the nearest related species with a genetic distance 6.15%; however, it is very far from *I.proximaria* on the NJ tree. It can be inferred that, on the basis of the public COI sequences available on BOLD, the closest species to *I.proximaria* has not yet been found.

In the present work, the main purpose of DNA barcoding is to identify whether *I.proximaria* has intraspecific variation; the results of NJ tree for a large number of species are not included, which is beyond the scope of this work, and only three sequences of *I.craspedota*, with similar wing patterns, are included (Fig. 15).

#### ﻿ Idaea rectangularis

Taxon classificationAnimaliaLepidopteraGeometridae

Cheng & Han sp. nov.

5E568580-6D75-5142-B626-CC5075C44EA4

https://zoobank.org/04A6A888-DC93-4F3F-BA65-F9605770D75F

Figs 8 , 9 , 20 , 27 , 31

##### Type material.

***Holotype***, ♂, **China: Guangxi** (IZCAS): Jinxiu, Linhaishanzhuang, 1000 m, 2.VII.2000, leg. Li Wenzhu, slide no. Geom-5160. ***Paratypes*: Guangxi** (IZCAS): 1♂2♀, same data as holotype, slide no. Geom-5161(♀); 1♀, Huanjiang, Yangmeiao, 1189 m, 18–22.VII.2015, leg. Jiang Nan, slide no. Geom-5187. **Fujian** (IZCAS): 1♀, Chongan, Xingcun, Guadun, 900–1160 m, 8.VII.1963, leg. Zhang Youwei; 1♀, Wuyishan, Dazhulan, 1150 m, 28.VII.2006, leg. Xue Dayong, slide no. Geom-5182.

##### Diagnosis.

Compared to *I.proximaria*, the forewing apex of *I.rectangularis* is somewhat sharper, and the first segment of male hind tarsus is darker. The male genitalia of *I.rectangularis* are similar to those of *I.proximaria*, but can be differentiated by the following characters: the anellus is smaller than that of *I.proximaria*, and the anterior half lacks spines on the lateral margin in *I.rectangularis* whereas these are present in *I.proximaria*; the valva possesses a small blunt finger-like process in *I.rectangularis*, whereas in *I.proximaria* there is only a protrusion which does not form a process ; the slightly concave uncus is also different. The aedeagus is distinguished by the number of the cornuti, which is five in *I.rectangularis*, including one right-angled spine, but only one or two in *I.proximaria*. In the female genitalia, the region surrounding the ostium is not totally sclerotised, with a separate lamella postvaginalis. The ductus bursae lacks a bag-like process in *I.rectangularis* but it is present in *I.proximaria*.

##### Description.

***Head*.** Antennae ciliate in male, filiform in female. Frons, labial palpus, vertex identical to *I.proximaria*.

***Thorax*.** Hind leg similar to that of *I.proximaria*, but first segment of male hind tarsus blackish grey. Forewing length: male 10 mm, female 10–11 mm. Wing pattern and markings similar to those of *I.proximaria*, except that the apex of the male forewing protrudes further.

***Abdomen*. *Male genitalia*.** Uncus spatulate, posterior margin slightly concave. Gnathos developed, tapering. Valva long and slender, with a sclerotised ridge at middle; apex rounded and setose; dorsal margin slightly concave at middle, with a faint subapical protuberance; ventral margin deeply concave near middle, with a small finger-like blunt process. Saccus blunt. Anellus a large sclerite, posterior half rounded, anterior half an inverted trapezoid. Aedeagus stout, terminal two-thirds sclerotised and broadened; cornutus with five spines, one larger than the rest and right-angled; vesica with a scobinate area.

***Female genitalia*.** Ovipositor lobes with a small ventral protrusion. Apophyses posteriores ~ 2× length of apophyses anteriores. Lamella postvaginalis a laterally elongate sclerite, wrinkled. Lamella antevaginalis shapeless, but with left side of ostium strongly sclerotised, scobinate anteriorly. Ductus bursae very broad, wrinkled, the large posterior half decorated with long spines, anteriorly slightly sclerotised; an elongate posteriorly diverging appendix bursae present. Corpus bursae small, rounded, decorated with an oval spinose patch.

##### Distribution.

China (Guangxi, Fujian).

##### Etymology.

This species is named from Latin word *rectangularis*, which refers to the one right-angled cornutus.

#### ﻿ Idaea rectispina

Taxon classificationAnimaliaLepidopteraGeometridae

Cheng & Han sp. nov.

4ADC1D6E-3ECD-5C99-A715-0E7919B27980

https://zoobank.org/05FC5D62-74D5-450A-B46D-148F15222896

Figs 10 , 21 , 28

##### Type material.

***Holotype***, ♂, **China: Hunan** (IZCAS): Yanling, Taoyuandong, 631 m, 4–8.VII.2008, leg. Chen Fuqiang, slide no. Geom-5159.

##### Diagnosis.

In the male genitalia, *I.rectispina* is similar to *I.rectangularis* in having a concave uncus, tongue-like gnathos, and in the presence of a small finger-like process on the ventral margin of the valva. However, small but differently sized protrusions are present on the valval costal margin of *I.rectispina*, but absent in *I.rectangularis*. The number of cornuti in the aedeagus is six and all are straight in *I.rectispina*, as opposed to the five in *I.rectangularis* with one right-angled.

##### Description.

Wing pattern almost identical to that of *I.proximaria*, *I.rectangularis*, and *I.setosa*. First segment of male hind tarsus covered with blackish scales. Forewing length: male 10 mm.

***Abdomen*. *Male genitalia*.** Uncus spatulate, posterior margin slightly concave. Gnathos developed, tongue-like, with tip narrow. Valva long and slender, apex rounded and setose; dorsal margin with basal half decorated with small protrusions of different sizes, asymmetrical on the left and right valvae; ventral margin concave at middle, with a small blunt finger-like process. Saccus narrow and blunt. Anellus a large sclerite. Aedeagus with terminal part broadened, cornutus with six straight spines, vesica also with a scobinate area.

***Female genitalia*.** Unknown.

##### Distribution.

China (Hunan).

##### Etymology.

This species is named from Latin prefix *rect*- and Latin word *spina*, referring to the straight spines of the cornutus.

#### ﻿ Idaea setosa

Taxon classificationAnimaliaLepidopteraGeometridae

Xue & Han sp. nov.

CD0A1F17-1EEA-5EE9-84AF-03EB524689B3

https://zoobank.org/4FA09D29-543F-4D58-AA69-90AA12C44FB2

Figs 11–12 , 22 , 29 , 32

##### Type material.

***Holotype***, ♂, **China: Hainan** (IZCAS): Qiongzhong, Limushan, Qijiacun, 657 m, 6–7.IV.2010, leg. Jiang Nan, slide no. Geom-5140. ***Paratypes*: Hainan** (IZCAS): 1♂1♀, Jianfengling, 900 m, 12.IV.1980, leg. Zhang Baolin; 1♂, Baisha, Yinggeling, Nankai, Mohaocun, 336 m, 15–16.IV.2010, leg. Jiang Nan; 1♂1♀, Bawangling, Dongerlinchang, 1015 m, 8–10.V.2007, leg. Chen Fuqiang, slide no. Geom-5141(♀).

##### Diagnosis.

The wing pattern is almost identical to that of *I.proximaria*, *I.rectangularis*, and *I.rectispina*. The first segment of the male hind tarsus is pure brown, which is different from the blackish hind tarsus in other species. In the male genitalia, *I.setosa* is distinctive in the *proximaria* complex by the uncus having a swollen posterior half, by the triangular gnathos, and by the valva having a subapical ventral spine. The aedeagus is even in width, not broadened posteriorly, and the cornutus has many more spines than in the other species. The female genitalia are distinctive with a very short ductus bursae and the large corpus bursae.

##### Description.

Forewing length: male 10 mm, female 9–12 mm. First segment of male hind tarsus pure brown. Head and wing pattern almost identical to *I.proximaria*, *I.rectangularis*, and *I.rectispina.* Forewing costa lacking dark purplish brown scales in females.

***Abdomen*. *Male genitalia*.** Uncus long, with posterior half swollen and rounded. Gnathos nearly triangular, with blunt tip. Valva long and slender, tapering, apex rounded and setose; dorsal margin nearly straight and only slightly convex near tip; ventral margin with basal two-thirds straight, concave near apex, bearing a finger-like blunt process. Saccus blunt. Anellus a large sclerite, posterior margin flat, slightly tapering towards anterior margin. Aedeagus in even width, cornutus a bunch of long spines, more than 30 spines.

***Female genitalia*.** Ovipositor lobes with small ventral protrusion. Apophyses posteriores ~ 2× length of apophyses anteriores. Region around ostium membranous, lamella postvaginalis and antevaginalis unmodified. Ductus bursae very short, a small pouch-like process present, an elongate diverticulum present. Corpus bursae very large and rounded, a rounded sclerotised patch present at middle of posterior half, bearing needle-like spines.

**Figures 28–34.**
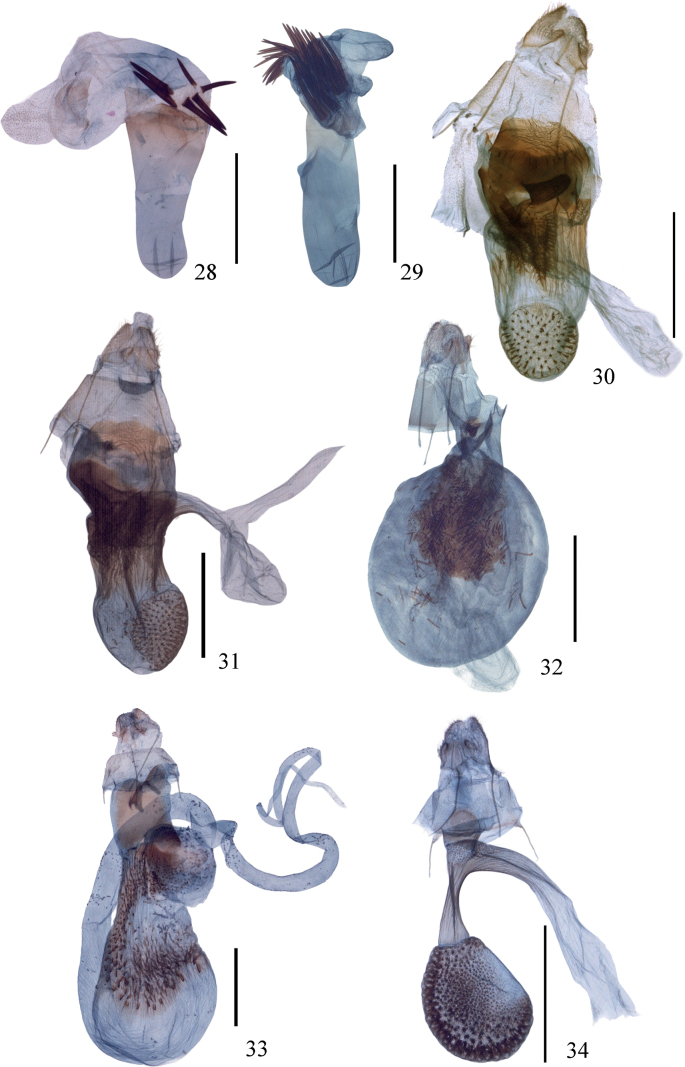
(**28–29**) Aedeagus of *I.proximaria* complex **28***I.rectispina* sp. nov., holotype **29***I.setosa* sp. nov., holotype (**30–34**) Female genitalia of *I.proximaria* complex **30***I.proximaria***31***I.rectangularis* sp. nov., paratype **32***I.setosa* sp. nov., paratype **33***I.linearis* sp. nov., holotype **34***I.craspedota*. Scale bars: 1 mm.

##### Distribution.

China (Hainan).

##### Etymology.

This species is named from Latin word *setosus*, referring to the bunch of long spines in the cornutus.

#### ﻿ Idaea linearis

Taxon classificationAnimaliaLepidopteraGeometridae

Xue & Han sp. nov.

A5B4DB23-2C4C-5D89-BFA9-CB2723A55EFE

https://zoobank.org/FE4AD323-9FBA-468D-BD76-E9BA05018C46

Figs 13 , 33

##### Type material.

***Holotype***, ♀, **China: Hubei** (IZCAS): Yingshan, Taohuachong, 590 m, 23–27.VI.2014, leg. Xue Dayong, slide no. Geom-5170. ***Paratypes*: Hubei** (IZCAS), 5♀, same data as holotype, leg. Jiang Nan et al., slide no. Geom-5169. **Shaanxi** (IZCAS): 1♀, Zhashui, Yingpanzhen, Niubeiliang, Laolin, 1046 m, 12–15.VII.2017, leg. Cui Le; 1♀, Foping, Yueba, 1093 m, 29.VI.–1.VII.2012, leg. Li Jing, slide no. Geom-5183; 1♀, Shangnan, Jinsixia, 766 m, 16–19.VII.2017, leg. Cui Le, slide no. Geom-5139.

##### Diagnosis.

On the wing pattern, *I.linearis* is distinctive in that the postmedial line is continuous and does not form black dots on the veins. The female genitalia are distinguished by the smooth lamella postvaginalis, which is divided posteriorly and bears a pair of processes; by the connection of the spinules of the ductus bursae and the corpus bursae, which form a spoon-shaped patch; and by the presence of the appendix bursae diverging anteriorly from the corpus bursae.

##### Description.

Head and wing patterns almost identical to previous species, except that the postmedial line is continuous rather than consisting of black spots on veins. Forewing length: female 10–11 mm. Forewing costa lacking dark purplish brown scales in female, unknown in male.

***Abdomen*. *Male genitalia*.** Unknown.

***Female genitalia*.** Ovipositor lobes with small ventral protrusion. Apophyses posteriores ~ 2× length of apophyses anteriores. Lamella postvaginalis an elongate smooth sclerite, posterior margin divided, with a pair of proximal processes. Lamella antevaginalis shapeless. Ductus bursae short, wrinkled and spinulose, with a small, rounded, appendix bursae. Corpus bursae nearly oval, lateral half spinulose and joined with spinules of ductus bursae, expanded into centre of corpus bursae, spoon-shaped; with a long, elongate anterior appendix bursae. Seventh sternite with a pair of small proximal bag-like protrusions.

##### Distribution.

China (Shaanxi, Hubei).

##### Etymology.

This species is named from the Latin word *linearis*, referring to the linear postmedial line.

## Supplementary Material

XML Treatment for Idaea proximaria

XML Treatment for Idaea rectangularis

XML Treatment for Idaea rectispina

XML Treatment for Idaea setosa

XML Treatment for Idaea linearis

## ﻿Additional information

### Author ORCIDs

Rui Cheng https://orcid.org/0000-0002-3044-4724

Da-Yong Xue https://orcid.org/0000-0002-5603-4459

Nan Jiang https://orcid.org/0000-0003-4211-1385

Hong-Xiang Han https://orcid.org/0000-0001-7538-2902

### Data availability

All of the data that support the findings of this study are available in the main text or Supplementary Information.

## Supplementary materials

Supplementary material 1 NJ tree of total *Idaea* species Data type Phylogenetic https://binary.pensoft.net/file/880635This dataset is made available under the Open Database License (http://opendatacommons.org/licenses/odbl/1.0/). The Open Database License (ODbL) is a license agreement intended to allow users to freely share, modify, and use this Dataset while maintaining this same freedom for others, provided that the original source and author(s) are credited.Rui Cheng, Da-Yong Xue, Nan Jiang, Hong-Xiang Han
