# Supplementary material for: ﻿Review of the Idaeaproximaria complex (Lepidoptera, Geometridae, Sterrhinae) with descriptions of four new species
Source: Zookeys. 2023 Jul 18;1169:293–305. doi: 10.3897/zookeys.1169.106815 (PMC10369171; doi:10.3897/zookeys.1169.106815)
Supplement: Supplementary material 1 — NJ tree of total Idaea species [file zookeys-1169-293_article-106815__-s001.pdf]

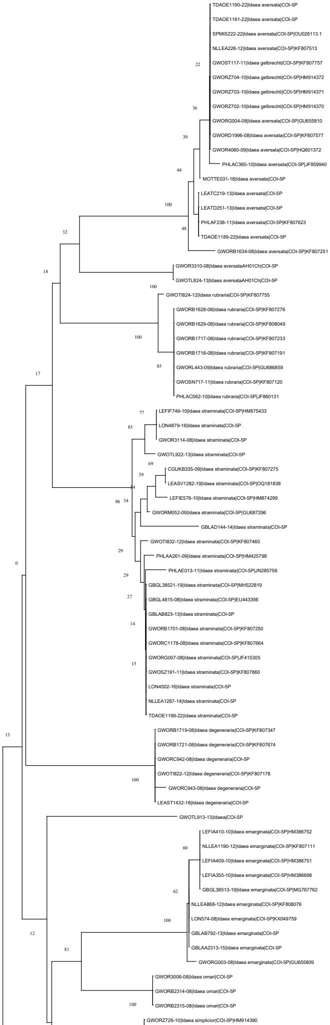
TDAOE1190-22|Idaea aversata|COI-5P
TDAOE1191-22|Idaea aversata|COI-5P
SPMIS222-22|Idaea aversata|COI-5P|OU026113.1
NLLEA226-12|Idaea aversata|COI-5P|KF807513
22
GWOST117-11|Idaea gelbrechti|COI-5P|KF807757
GWORZ704-10|Idaea gelbrechti|COI-5P|HM914372
GWORZ703-10|Idaea gelbrechti|COI-5P|HM914371
36
GWORZ702-10|Idaea gelbrechti|COI-5P|HM914370
GWORG004-08|Idaea aversata|COI-5P|GU655810
39
GWORD1996-08|Idaea aversata|COI-5P|KF807577
GWOR4060-09|Idaea aversata|COI-5P|HQ601372
PHLAC360-10|Idaea aversata|COI-5P|JF859940
44
MOTTE031-18|Idaea aversata|COI-5P
LEATC219-13|Idaea aversata|COI-5P
100
LEATD251-13|Idaea aversata|COI-5P
PHLAF238-11|Idaea aversata|COI-5P|KF807623
48
32
TDAOE1189-22|Idaea aversata|COI-5P
GWORB1634-08|Idaea aversata|COI-5P|KF807251
18
GWOR3310-08|Idaea aversataAH01Ch|COI-5P
GWOTL824-13|Idaea aversataAH01Ch|COI-5P
100
GWOTI824-12|Idaea rubraria|COI-5P|KF807755
GWORB1628-08|Idaea rubraria|COI-5P|KF807276
GWORB1629-08|Idaea rubraria|COI-5P|KF808049
100
GWORB1717-08|Idaea rubraria|COI-5P|KF807233
GWORB1718-08|Idaea rubraria|COI-5P|KF807191
85
GWORL443-09|Idaea rubraria|COI-5P|GU686859
17
GWOSN717-11|Idaea rubraria|COI-5P|KF807120
PHLAC562-10|Idaea rubraria|COI-5P|JF860131
77
LEFIF749-10|Idaea straminata|COI-5P|HM875433
85
LON4879-16|Idaea straminata|COI-5P
GWOR3114-08|Idaea straminata|COI-5P
GWOTL922-13|Idaea straminata|COI-5P
69
CGUKB335-09|Idaea straminata|COI-5P|KF807275
39
LEASV1282-19|Idaea straminata|COI-5P|OQ181838
24
LEFIE576-10|Idaea straminata|COI-5P|HM874299
98
34
GWORM052-09|Idaea straminata|COI-5P|GU687296
GBLAD144-14|Idaea straminata|COI-5P
GWOTI832-12|Idaea straminata|COI-5P|KF807460
29
PHLAA261-09|Idaea straminata|COI-5P|HM425798
0
PHLAE013-11|Idaea straminata|COI-5P|JN285756
29
GBGL38521-19|Idaea straminata|COI-5P|MH522819
27
GBGL4815-08|Idaea straminata|COI-5P|EU443356
GBLAB823-13|Idaea straminata|COI-5P
14
GWORB1701-08|Idaea straminata|COI-5P|KF807250
GWORC1178-08|Idaea straminata|COI-5P|KF807664
GWORG007-08|Idaea straminata|COI-5P|JF415305
15
GWOSZ191-11|Idaea straminata|COI-5P|KF807860
LON4502-16|Idaea straminata|COI-5P
NLLEA1287-14|Idaea straminata|COI-5P
TDAOE1199-22|Idaea straminata|COI-5P
GWORB1719-08|Idaea degeneraria|COI-5P|KF807347
13
GWORB1721-08|Idaea degeneraria|COI-5P|KF807674
GWORC942-08|Idaea degeneraria|COI-5P
GWOTI822-12|Idaea degeneraria|COI-5P|KF807178
100
GWORC943-08|Idaea degeneraria|COI-5P
LEAST1432-18|Idaea degeneraria|COI-5P
GWOTL913-13|Idaea|COI-5P
LEFIA410-10|Idaea emarginata|COI-5P|HM386752
60
NLLEA1190-12|Idaea emarginata|COI-5P|KF807111
LEFIA409-10|Idaea emarginata|COI-5P|HM386751
LEFIA355-10|Idaea emarginata|COI-5P|HM386698
GBGL38513-19|Idaea emarginata|COI-5P|MG767762
62
NLLEA868-12|Idaea emarginata|COI-5P|KF808076
LON574-08|Idaea emarginata|COI-5P|KX049759
100
12
GBLAB792-13|Idaea emarginata|COI-5P
81
GBLAA2313-15|Idaea emarginata|COI-5P
GWORG003-08|Idaea emarginata|COI-5P|GU655809
GWOR3006-08|Idaea omari|COI-5P
GWORB2314-08|Idaea omari|COI-5P
100
GWORB2315-08|Idaea omari|COI-5P
GWORZ726-10|Idaea simplicior|COI-5P|HM914390

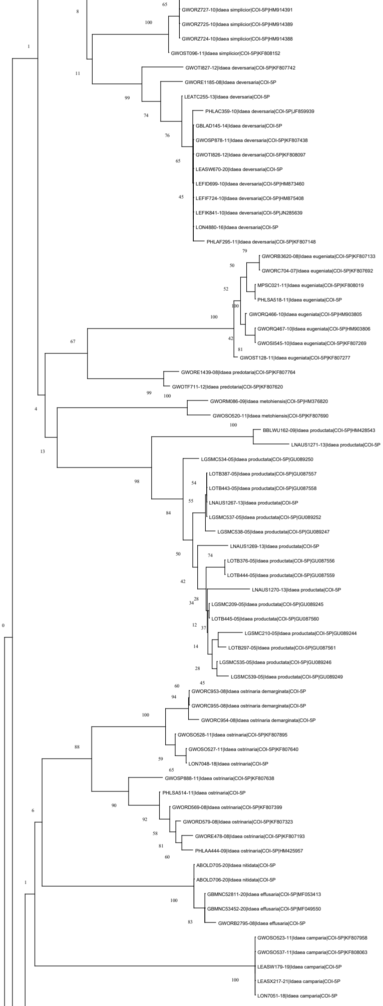
GWORZ727-10|Idaea simplicior|COI-5P|HM914391
GWORZ725-10|Idaea simplicior|COI-5P|HM914389
GWORZ724-10|Idaea simplicior|COI-5P|HM914388
GWOST096-11|Idaea simplicior|COI-5P|KF808152
GWOTI827-12|Idaea deversaria|COI-5P|KF807742
GWORE1185-08|Idaea deversaria|COI-5P
LEATC255-13|Idaea deversaria|COI-5P
PHLAC359-10|Idaea deversaria|COI-5P|JF859939
GBLAD145-14|Idaea deversaria|COI-5P
GWOSP878-11|Idaea deversaria|COI-5P|KF807438
GWOTI826-12|Idaea deversaria|COI-5P|KF808097
LEASW670-20|Idaea deversaria|COI-5P
LEFID699-10|Idaea deversaria|COI-5P|HM873460
LEFIF724-10|Idaea deversaria|COI-5P|HM875408
LEFIK841-10|Idaea deversaria|COI-5P|JN285639
LON4880-16|Idaea deversaria|COI-5P
PHLAF295-11|Idaea deversaria|COI-5P|KF807148
GWORB3620-08|Idaea eugeniata|COI-5P|KF807133
GWORC704-07|Idaea eugeniata|COI-5P|KF807692
MPSC021-11|Idaea eugeniata|COI-5P|KF808019
PHLSA518-11|Idaea eugeniata|COI-5P
GWORQ466-10|Idaea eugeniata|COI-5P|HM903805
GWORQ467-10|Idaea eugeniata|COI-5P|HM903806
GWOSI545-10|Idaea eugeniata|COI-5P|KF807269
GWOST128-11|Idaea eugeniata|COI-5P|KF807277
GWORE1439-08|Idaea predotaria|COI-5P|KF807764
GWOTF711-12|Idaea predotaria|COI-5P|KF807620
GWORM086-09|Idaea metohiensis|COI-5P|HM376820
GWOSO520-11|Idaea metohiensis|COI-5P|KF807690
BBLWU162-09|Idaea productata|COI-5P|HM428543
LNAUS1271-13|Idaea productata|COI-5P
LGSMC534-05|Idaea productata|COI-5P|GU089250
LOTB387-05|Idaea productata|COI-5P|GU087557
LOTB443-05|Idaea productata|COI-5P|GU087558
LNAUS1267-13|Idaea productata|COI-5P
LGSMC537-05|Idaea productata|COI-5P|GU089252
LGSMC538-05|Idaea productata|COI-5P|GU089247
LNAUS1269-13|Idaea productata|COI-5P
LOTB376-05|Idaea productata|COI-5P|GU087556
LOTB444-05|Idaea productata|COI-5P|GU087559
LNAUS1270-13|Idaea productata|COI-5P
LGSMC209-05|Idaea productata|COI-5P|GU089245
LOTB445-05|Idaea productata|COI-5P|GU087560
LGSMC210-05|Idaea productata|COI-5P|GU089244
LOTB297-05|Idaea productata|COI-5P|GU087561
LGSMC535-05|Idaea productata|COI-5P|GU089246
LGSMC539-05|Idaea productata|COI-5P|GU089249
GWORC953-08|Idaea ostrinaria demarginata|COI-5P
GWORC955-08|Idaea ostrinaria demarginata|COI-5P
GWORC954-08|Idaea ostrinaria demarginata|COI-5P
GWOSO528-11|Idaea ostrinaria|COI-5P|KF807895
GWOSO527-11|Idaea ostrinaria|COI-5P|KF807640
LON7048-18|Idaea ostrinaria|COI-5P
GWOSP888-11|Idaea ostrinaria|COI-5P|KF807638
PHLSA514-11|Idaea ostrinaria|COI-5P
GWORD569-08|Idaea ostrinaria|COI-5P|KF807399
GWORD579-08|Idaea ostrinaria|COI-5P|KF807323
GWORE478-08|Idaea ostrinaria|COI-5P|KF807193
PHLAA444-09|Idaea ostrinaria|COI-5P|HM425957
ABOLD705-20|Idaea nitidata|COI-5P
ABOLD706-20|Idaea nitidata|COI-5P
GBMNC52811-20|Idaea effusaria|COI-5P|MF053413
GBMNC53452-20|Idaea effusaria|COI-5P|MF049550
GWORB2795-08|Idaea effusaria|COI-5P
GWOSO523-11|Idaea camparia|COI-5P|KF807958
GWOSO537-11|Idaea camparia|COI-5P|KF808063
LEASW179-19|Idaea camparia|COI-5P
LEASX217-21|Idaea camparia|COI-5P
LON7051-18|Idaea camparia|COI-5P

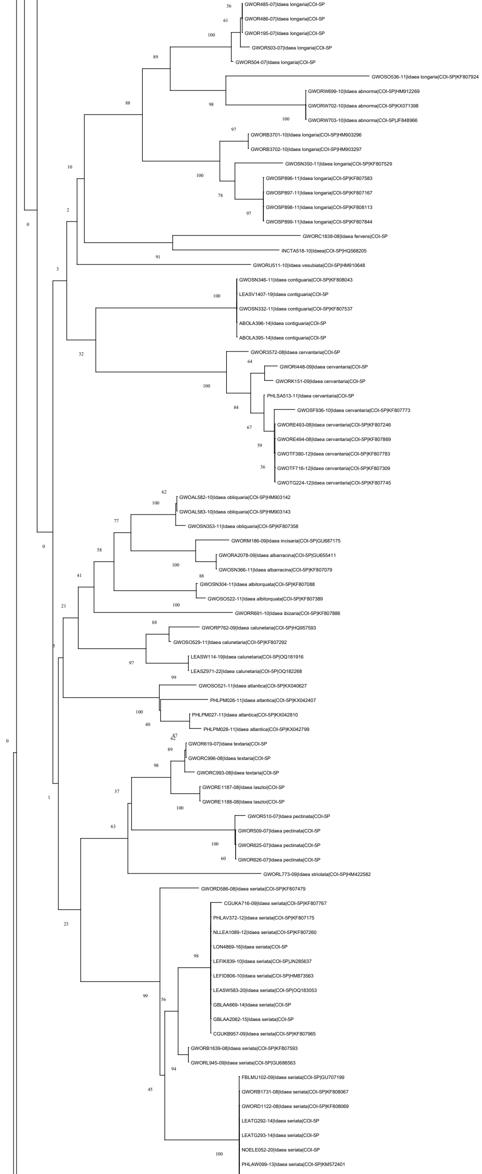
GWOR485-07|Idaea longaria|COI-5P
GWOR486-07|Idaea longaria|COI-5P
GWOR195-07|Idaea longaria|COI-5P
GWOR503-07|Idaea longaria|COI-5P
GWOR504-07|Idaea longaria|COI-5P
GWOSO536-11|Idaea longaria|COI-5P|KF807924
GWORW699-10|Idaea abnorma|COI-5P|HM912269
GWORW702-10|Idaea abnorma|COI-5P|KX071398
GWORW703-10|Idaea abnorma|COI-5P|JF848966
GWORB3701-10|Idaea longaria|COI-5P|HM903296
GWORB3702-10|Idaea longaria|COI-5P|HM903297
GWOSN350-11|Idaea longaria|COI-5P|KF807529
GWOSP896-11|Idaea longaria|COI-5P|KF807583
GWOSP897-11|Idaea longaria|COI-5P|KF807167
GWOSP898-11|Idaea longaria|COI-5P|KF808113
GWOSP899-11|Idaea longaria|COI-5P|KF807844
GWORC1838-08|Idaea fervens|COI-5P
INCTA518-10|Idaea|COI-5P|HQ568205
GWORU511-10|Idaea vesubiata|COI-5P|HM910648
GWOSN346-11|Idaea contiguaria|COI-5P|KF808043
LEASV1407-19|Idaea contiguaria|COI-5P
GWOSN332-11|Idaea contiguaria|COI-5P|KF807537
ABOLA396-14|Idaea contiguaria|COI-5P
ABOLA395-14|Idaea contiguaria|COI-5P
GWOR3572-08|Idaea cervantaria|COI-5P
GWORI448-09|Idaea cervantaria|COI-5P
GWORK151-09|Idaea cervantaria|COI-5P
PHLSA513-11|Idaea cervantaria|COI-5P
GWOSF936-10|Idaea cervantaria|COI-5P|KF807773
GWORE493-08|Idaea cervantaria|COI-5P|KF807246
GWORE494-08|Idaea cervantaria|COI-5P|KF807869
GWOTF380-12|Idaea cervantaria|COI-5P|KF807783
GWOTF716-12|Idaea cervantaria|COI-5P|KF807309
GWOTG224-12|Idaea cervantaria|COI-5P|KF807745
GWOAL582-10|Idaea obliquaria|COI-5P|HM903142
GWOAL583-10|Idaea obliquaria|COI-5P|HM903143
GWOSN353-11|Idaea obliquaria|COI-5P|KF807358
GWORM186-09|Idaea incisaria|COI-5P|GU687175
GWORA2078-09|Idaea albarracina|COI-5P|GU655411
GWOSN366-11|Idaea albarracina|COI-5P|KF807079
GWOSN304-11|Idaea albitorquata|COI-5P|KF807088
GWOSO522-11|Idaea albitorquata|COI-5P|KF807389
GWORR691-10|Idaea ibizaria|COI-5P|KF807886
GWORP762-09|Idaea calunetaria|COI-5P|HQ957593
GWOSO529-11|Idaea calunetaria|COI-5P|KF807292
LEASW114-19|Idaea calunetaria|COI-5P|OQ181916
LEASZ971-22|Idaea calunetaria|COI-5P|OQ182268
GWOSO521-11|Idaea atlantica|COI-5P|KX040627
PHLPM026-11|Idaea atlantica|COI-5P|KX042407
PHLPM027-11|Idaea atlantica|COI-5P|KX042810
PHLPM028-11|Idaea atlantica|COI-5P|KX042799
GWOR619-07|Idaea textaria|COI-5P
GWORC996-08|Idaea textaria|COI-5P
GWORC993-08|Idaea textaria|COI-5P
GWORE1187-08|Idaea laszloi|COI-5P
GWORE1188-08|Idaea laszloi|COI-5P
GWOR510-07|Idaea pectinata|COI-5P
GWOR509-07|Idaea pectinata|COI-5P
GWOR625-07|Idaea pectinata|COI-5P
GWOR626-07|Idaea pectinata|COI-5P
GWORL773-09|Idaea striolata|COI-5P|HM422582
GWORD586-08|Idaea seriata|COI-5P|KF807479
CGUKA716-09|Idaea seriata|COI-5P|KF807767
PHLAV372-12|Idaea seriata|COI-5P|KF807175
NLLEA1089-12|Idaea seriata|COI-5P|KF807260
LON4869-16|Idaea seriata|COI-5P
LEFIK839-10|Idaea seriata|COI-5P|JN285637
LEFID806-10|Idaea seriata|COI-5P|HM873563
LEASW583-20|Idaea seriata|COI-5P|OQ183053
GBLAA669-14|Idaea seriata|COI-5P
GBLAA2062-15|Idaea seriata|COI-5P
CGUKB957-09|Idaea seriata|COI-5P|KF807965
GWORB1639-08|Idaea seriata|COI-5P|KF807593
GWORL945-09|Idaea seriata|COI-5P|GU686563
FBLMU102-09|Idaea seriata|COI-5P|GU707199
GWORB1731-08|Idaea seriata|COI-5P|KF808067
GWORD1122-08|Idaea seriata|COI-5P|KF808069
LEATG292-14|Idaea seriata|COI-5P
LEATG293-14|Idaea seriata|COI-5P
NOELE052-20|Idaea seriata|COI-5P
PHLAW099-13|Idaea seriata|COI-5P|KM572401

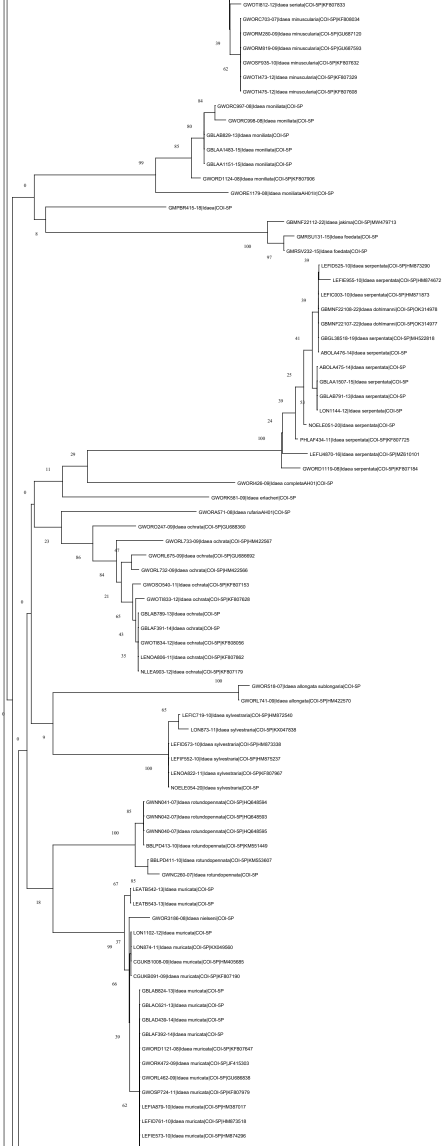
GWOTI812-12|Idaea seriata|COI-5P|KF807833
GWORC703-07|Idaea minuscularia|COI-5P|KF808034
GWORM280-09|Idaea minuscularia|COI-5P|GU687120
39
GWORM819-09|Idaea minuscularia|COI-5P|GU687593
62
GWOSF935-10|Idaea minuscularia|COI-5P|KF807632
GWOTI473-12|Idaea minuscularia|COI-5P|KF807329
GWOTI475-12|Idaea minuscularia|COI-5P|KF807608
84
GWORC997-08|Idaea moniliata|COI-5P
80
GWORC998-08|Idaea moniliata|COI-5P
GBLAB829-13|Idaea moniliata|COI-5P
85
GBLAA1483-15|Idaea moniliata|COI-5P
99
GBLAA1151-15|Idaea moniliata|COI-5P
0
GWORD1124-08|Idaea moniliata|COI-5P|KF807906
GWORE1179-08|Idaea moniliataAH01Ir|COI-5P
GMPBR415-18|Idaea|COI-5P
8
GBMNF22112-22|Idaea jakima|COI-5P|MW479713
GMRSU131-15|Idaea foedata|COI-5P
100
GMRSV232-15|Idaea foedata|COI-5P
97
39
LEFID525-10|Idaea serpentata|COI-5P|HM873290
LEFIE955-10|Idaea serpentata|COI-5P|HM874672
LEFIC003-10|Idaea serpentata|COI-5P|HM871873
39
GBMNF22108-22|Idaea dohlmanni|COI-5P|OK314978
GBMNF22107-22|Idaea dohlmanni|COI-5P|OK314977
41
GBGL38518-19|Idaea serpentata|COI-5P|MH522818
ABOLA476-14|Idaea serpentata|COI-5P
ABOLA475-14|Idaea serpentata|COI-5P
25
GBLAA1507-15|Idaea serpentata|COI-5P
39
GBLAB791-13|Idaea serpentata|COI-5P
53
LON1144-12|Idaea serpentata|COI-5P
24
NOELE051-20|Idaea serpentata|COI-5P
100
PHLAF434-11|Idaea serpentata|COI-5P|KF807725
29
LEFIJ4870-16|Idaea serpentata|COI-5P|MZ610101
11
GWORD1119-08|Idaea serpentata|COI-5P|KF807184
GWORI426-09|Idaea completaAH01|COI-5P
0
GWORK581-09|Idaea erlacheri|COI-5P
GWORA571-08|Idaea rufariaAH01|COI-5P
GWORO247-09|Idaea ochrata|COI-5P|GU688360
23
GWORL733-09|Idaea ochrata|COI-5P|HM422567
86
47
GWORL675-09|Idaea ochrata|COI-5P|GU686692
84
GWORL732-09|Idaea ochrata|COI-5P|HM422566
GWOSO540-11|Idaea ochrata|COI-5P|KF807153
21
GWOTI833-12|Idaea ochrata|COI-5P|KF807628
0
65
GBLAB789-13|Idaea ochrata|COI-5P
GBLAF391-14|Idaea ochrata|COI-5P
43
GWOTI834-12|Idaea ochrata|COI-5P|KF808056
35
LENOA806-11|Idaea ochrata|COI-5P|KF807862
NLLEA903-12|Idaea ochrata|COI-5P|KF807179
100
GWOR518-07|Idaea allongata sublongaria|COI-5P
GWORL741-09|Idaea allongata|COI-5P|HM422570
65
LEFIC719-10|Idaea sylvestraria|COI-5P|HM872540
0
LON873-11|Idaea sylvestraria|COI-5P|KX047838
0
9
LEFID573-10|Idaea sylvestraria|COI-5P|HM873338
LEFIF552-10|Idaea sylvestraria|COI-5P|HM875237
100
LENOA822-11|Idaea sylvestraria|COI-5P|KF807967
NOELE054-20|Idaea sylvestraria|COI-5P
GWNN041-07|Idaea rotundopennata|COI-5P|HQ648594
85
GWNN042-07|Idaea rotundopennata|COI-5P|HQ648593
100
GWNN040-07|Idaea rotundopennata|COI-5P|HQ648595
BBLPD413-10|Idaea rotundopennata|COI-5P|KM551449
BBLPD411-10|Idaea rotundopennata|COI-5P|KM553607
GWNC260-07|Idaea rotundopennata|COI-5P
67
85
LEATB542-13|Idaea muricata|COI-5P
18
LEATB543-13|Idaea muricata|COI-5P
GWOR3186-08|Idaea nielseni|COI-5P
LON1102-12|Idaea muricata|COI-5P
37
99
LON874-11|Idaea muricata|COI-5P|KX049560
CGUKB1008-09|Idaea muricata|COI-5P|HM405685
CGUKB091-09|Idaea muricata|COI-5P|KF807190
66
GBLAB824-13|Idaea muricata|COI-5P
GBLAC621-13|Idaea muricata|COI-5P
GBLAD439-14|Idaea muricata|COI-5P
39
GBLAF392-14|Idaea muricata|COI-5P
GWORD1121-08|Idaea muricata|COI-5P|KF807647
GWORK472-09|Idaea muricata|COI-5P|JF415303
GWORL462-09|Idaea muricata|COI-5P|GU686838
GWOSP724-11|Idaea muricata|COI-5P|KF807979
62
LEFIA879-10|Idaea muricata|COI-5P|HM387017
LEFID761-10|Idaea muricata|COI-5P|HM873518
LEFIE573-10|Idaea muricata|COI-5P|HM874296

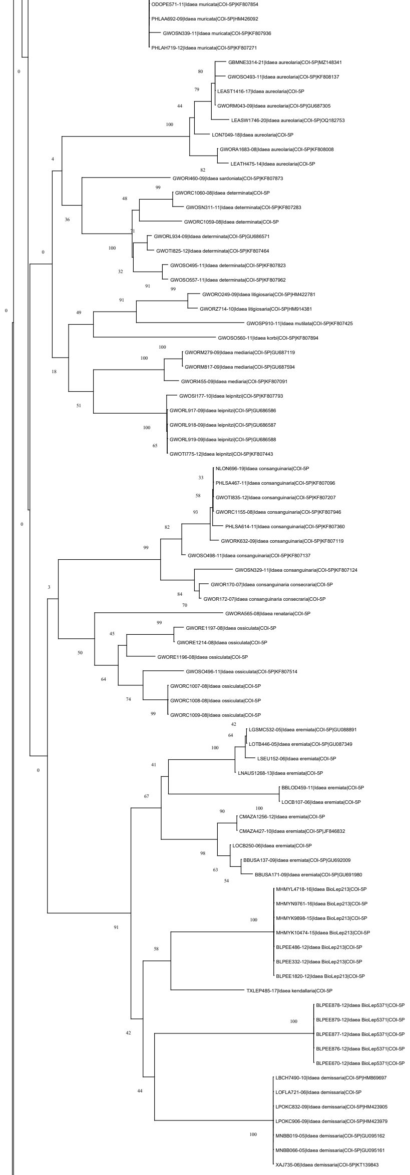

ODOPE571-11|Idaea muricata|COI-5P|KF807854
PHLAA692-09|Idaea muricata|COI-5P|HM426092
GWOSN339-11|Idaea muricata|COI-5P|KF807936
PHLAH719-12|Idaea muricata|COI-5P|KF807271
GBMNE3314-21|Idaea aureolaria|COI-5P|MZ148341
GWOSO493-11|Idaea aureolaria|COI-5P|KF808137
LEAST1416-17|Idaea aureolaria|COI-5P
GWORM043-09|Idaea aureolaria|COI-5P|GU687305
LEASW1746-20|Idaea aureolaria|COI-5P|OQ182753
LON7049-18|Idaea aureolaria|COI-5P
GWORA1683-08|Idaea aureolaria|COI-5P|KF808008
LEATH475-14|Idaea aureolaria|COI-5P
GWORI460-09|Idaea sardoniata|COI-5P|KF807873
GWORC1060-08|Idaea determinata|COI-5P
GWOSN311-11|Idaea determinata|COI-5P|KF807283
GWORC1059-08|Idaea determinata|COI-5P
GWORL934-09|Idaea determinata|COI-5P|GU686571
GWOTI825-12|Idaea determinata|COI-5P|KF807464
GWOSO495-11|Idaea determinata|COI-5P|KF807823
GWOSO557-11|Idaea determinata|COI-5P|KF807962
GWORO249-09|Idaea litigiosaria|COI-5P|HM422781
GWORZ714-10|Idaea litigiosaria|COI-5P|HM914381
GWOSP910-11|Idaea mutilata|COI-5P|KF807425
GWOSO560-11|Idaea korbi|COI-5P|KF807894
GWORM279-09|Idaea mediaria|COI-5P|GU687119
GWORM817-09|Idaea mediaria|COI-5P|GU687594
GWORI455-09|Idaea mediaria|COI-5P|KF807091
GWOSI177-10|Idaea leipnitzi|COI-5P|KF807793
GWORL917-09|Idaea leipnitzi|COI-5P|GU686586
GWORL918-09|Idaea leipnitzi|COI-5P|GU686587
GWORL919-09|Idaea leipnitzi|COI-5P|GU686588
GWOTI775-12|Idaea leipnitzi|COI-5P|KF807443
NLON696-19|Idaea consanguinaria|COI-5P
PHLSA467-11|Idaea consanguinaria|COI-5P|KF807096
GWOTI835-12|Idaea consanguinaria|COI-5P|KF807207
GWORC1155-08|Idaea consanguinaria|COI-5P|KF807946
PHLSA614-11|Idaea consanguinaria|COI-5P|KF807360
GWORK632-09|Idaea consanguinaria|COI-5P|KF807119
GWOSO498-11|Idaea consanguinaria|COI-5P|KF807137
GWOSN329-11|Idaea consanguinaria|COI-5P|KF807124
GWOR170-07|Idaea consanguinaria consecraria|COI-5P
GWOR172-07|Idaea consanguinaria consecraria|COI-5P
GWORA565-08|Idaea renataria|COI-5P
GWORE1197-08|Idaea ossiculata|COI-5P
GWORE1214-08|Idaea ossiculata|COI-5P
GWORE1196-08|Idaea ossiculata|COI-5P
GWOSO496-11|Idaea ossiculata|COI-5P|KF807514
GWORC1007-08|Idaea ossiculata|COI-5P
GWORC1008-08|Idaea ossiculata|COI-5P
GWORC1009-08|Idaea ossiculata|COI-5P
LGSMC532-05|Idaea eremiata|COI-5P|GU088891
LOTB446-05|Idaea eremiata|COI-5P|GU087349
LSEU152-06|Idaea eremiata|COI-5P
LNAUS1268-13|Idaea eremiata|COI-5P
BBLOD459-11|Idaea eremiata|COI-5P
LOCB107-06|Idaea eremiata|COI-5P
CMAZA1256-12|Idaea eremiata|COI-5P
CMAZA427-10|Idaea eremiata|COI-5P|JF846832
LOCB250-06|Idaea eremiata|COI-5P
BBUSA137-09|Idaea eremiata|COI-5P|GU692009
BBUSA171-09|Idaea eremiata|COI-5P|GU691980
MHMYL4718-16|Idaea BioLep213|COI-5P
MHMYN9761-16|Idaea BioLep213|COI-5P
MHMYK9898-15|Idaea BioLep213|COI-5P
MHMYK10474-15|Idaea BioLep213|COI-5P
BLPEE486-12|Idaea BioLep213|COI-5P
BLPEE332-12|Idaea BioLep213|COI-5P
BLPEE1820-12|Idaea BioLep213|COI-5P
TXLEP485-17|Idaea kendallaria|COI-5P
BLPEE878-12|Idaea BioLep5371|COI-5P
BLPEE879-12|Idaea BioLep5371|COI-5P
BLPEE877-12|Idaea BioLep5371|COI-5P
BLPEE876-12|Idaea BioLep5371|COI-5P
BLPEE670-12|Idaea BioLep5371|COI-5P
LBCH7490-10|Idaea demissaria|COI-5P|HM869697
LOFLA721-06|Idaea demissaria|COI-5P
LPOKC832-09|Idaea demissaria|COI-5P|HM423905
LPOKC906-09|Idaea demissaria|COI-5P|HM423979
MNBB019-05|Idaea demissaria|COI-5P|GU095162
MNBB066-05|Idaea demissaria|COI-5P|GU095161
XAJ735-06|Idaea demissaria|COI-5P|KT139843

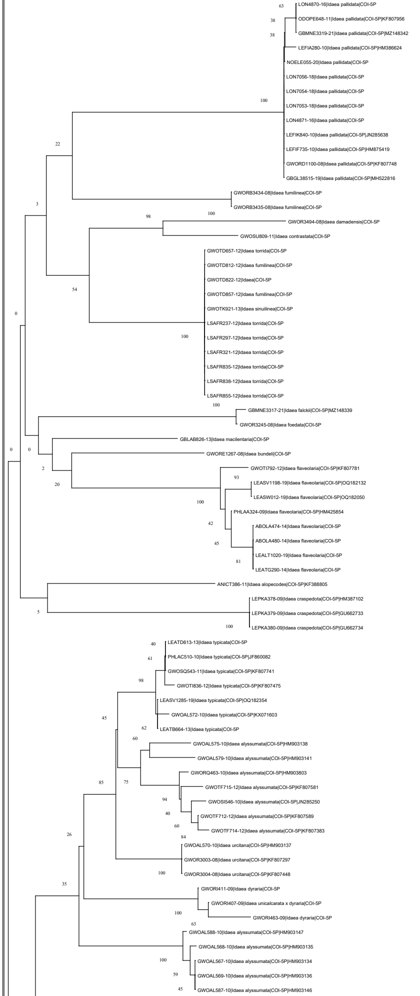
LON4870-16|Idaea pallidata|COI-5P
ODOPE648-11|Idaea pallidata|COI-5P|KF807956
GBMNE3319-21|Idaea pallidata|COI-5P|MZ148342
LEFIA280-10|Idaea pallidata|COI-5P|HM386624
NOELE055-20|Idaea pallidata|COI-5P
LON7056-18|Idaea pallidata|COI-5P
LON7054-18|Idaea pallidata|COI-5P
LON7053-18|Idaea pallidata|COI-5P
LON4871-16|Idaea pallidata|COI-5P
LEFIK840-10|Idaea pallidata|COI-5P|JN285638
LEFIF735-10|Idaea pallidata|COI-5P|HM875419
GWORD1100-08|Idaea pallidata|COI-5P|KF807748
GBGL38515-19|Idaea pallidata|COI-5P|MH522816
GWORB3434-08|Idaea fumilinea|COI-5P
GWORB3435-08|Idaea fumilinea|COI-5P
GWOR3494-08|Idaea damadensis|COI-5P
GWOSU809-11|Idaea contrastata|COI-5P
GWOTD657-12|Idaea torrida|COI-5P
GWOTD812-12|Idaea fumilinea|COI-5P
GWOTD822-12|Idaea|COI-5P
GWOTD857-12|Idaea fumilinea|COI-5P
GWOTK921-13|Idaea sinuilinea|COI-5P
LSAFR237-12|Idaea torrida|COI-5P
LSAFR297-12|Idaea torrida|COI-5P
LSAFR321-12|Idaea torrida|COI-5P
LSAFR835-12|Idaea torrida|COI-5P
LSAFR838-12|Idaea torrida|COI-5P
LSAFR855-12|Idaea torrida|COI-5P
GBMNE3317-21|Idaea falckii|COI-5P|MZ148339
GWOR3245-08|Idaea foedata|COI-5P
GBLAB826-13|Idaea macilentaria|COI-5P
GWORE1267-08|Idaea bundeli|COI-5P
GWOTI792-12|Idaea flaveolaria|COI-5P|KF807781
LEASV1198-19|Idaea flaveolaria|COI-5P|OQ182132
LEASW012-19|Idaea flaveolaria|COI-5P|OQ182050
PHLAA324-09|Idaea flaveolaria|COI-5P|HM425854
ABOLA474-14|Idaea flaveolaria|COI-5P
ABOLA480-14|Idaea flaveolaria|COI-5P
LEALT1020-19|Idaea flaveolaria|COI-5P
LEATG290-14|Idaea flaveolaria|COI-5P
ANICT386-11|Idaea alopecodes|COI-5P|KF388805
LEPKA378-09|Idaea craspedota|COI-5P|HM387102
LEPKA379-09|Idaea craspedota|COI-5P|GU662733
LEPKA380-09|Idaea craspedota|COI-5P|GU662734
LEATD613-13|Idaea typicata|COI-5P
PHLAC510-10|Idaea typicata|COI-5P|JF860082
GWOSQ543-11|Idaea typicata|COI-5P|KF807741
GWOTI836-12|Idaea typicata|COI-5P|KF807475
LEASV1285-19|Idaea typicata|COI-5P|OQ182354
GWOAL572-10|Idaea typicata|COI-5P|KX071603
LEATB664-13|Idaea typicata|COI-5P
GWOAL575-10|Idaea alyssumata|COI-5P|HM903138
GWOAL579-10|Idaea alyssumata|COI-5P|HM903141
GWORQ463-10|Idaea alyssumata|COI-5P|HM903803
GWOTF715-12|Idaea alyssumata|COI-5P|KF807581
GWOSI546-10|Idaea alyssumata|COI-5P|JN285250
GWOTF712-12|Idaea alyssumata|COI-5P|KF807589
GWOTF714-12|Idaea alyssumata|COI-5P|KF807383
GWOAL570-10|Idaea urcitana|COI-5P|HM903137
GWOR3003-08|Idaea urcitana|COI-5P|KF807297
GWOR3004-08|Idaea urcitana|COI-5P|KF807448
GWORI411-09|Idaea dyraria|COI-5P
GWORI407-09|Idaea unicalcarata x dyraria|COI-5P
GWORI463-09|Idaea dyraria|COI-5P
GWOAL588-10|Idaea alyssumata|COI-5P|HM903147
GWOAL568-10|Idaea alyssumata|COI-5P|HM903135
GWOAL567-10|Idaea alyssumata|COI-5P|HM903134
GWOAL569-10|Idaea alyssumata|COI-5P|HM903136
GWOAL587-10|Idaea alyssumata|COI-5P|HM903146

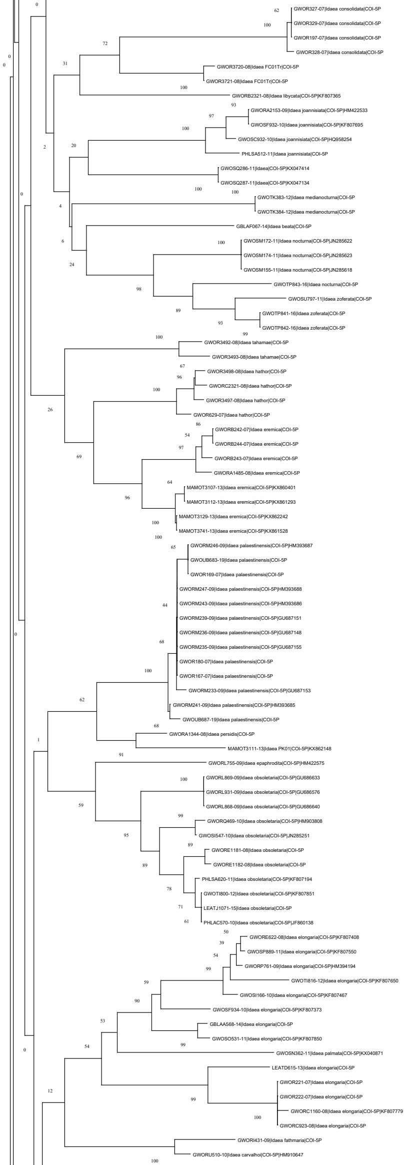
GWOR327-07|Idaea consolidata|COI-5P
GWOR329-07|Idaea consolidata|COI-5P
GWOR197-07|Idaea consolidata|COI-5P
GWOR328-07|Idaea consolidata|COI-5P
GWOR3720-08|Idaea FC01Tr|COI-5P
GWOR3721-08|Idaea FC01Tr|COI-5P
GWORB2321-08|Idaea libycata|COI-5P|KF807365
GWORA2153-09|Idaea joannisiata|COI-5P|HM422533
GWOSF932-10|Idaea joannisiata|COI-5P|KF807695
GWOSC932-10|Idaea joannisiata|COI-5P|HQ958254
PHLSA512-11|Idaea joannisiata|COI-5P
GWOSQ286-11|Idaea|COI-5P|KX047414
GWOSQ287-11|Idaea|COI-5P|KX047134
GWOTK383-12|Idaea medianocturna|COI-5P
GWOTK384-12|Idaea medianocturna|COI-5P
GBLAF067-14|Idaea beata|COI-5P
GWOSM172-11|Idaea nocturna|COI-5P|JN285622
GWOSM174-11|Idaea nocturna|COI-5P|JN285623
GWOSM155-11|Idaea nocturna|COI-5P|JN285618
GWOTP843-16|Idaea nocturna|COI-5P
GWOSU797-11|Idaea zoferata|COI-5P
GWOTP841-16|Idaea zoferata|COI-5P
GWOTP842-16|Idaea zoferata|COI-5P
GWOR3492-08|Idaea tahamae|COI-5P
GWOR3493-08|Idaea tahamae|COI-5P
GWOR3498-08|Idaea hathor|COI-5P
GWORC2321-08|Idaea hathor|COI-5P
GWOR3497-08|Idaea hathor|COI-5P
GWOR629-07|Idaea hathor|COI-5P
GWORB242-07|Idaea eremica|COI-5P
GWORB244-07|Idaea eremica|COI-5P
GWORB243-07|Idaea eremica|COI-5P
GWORA1485-08|Idaea eremica|COI-5P
MAMOT3107-13|Idaea eremica|COI-5P|KX860401
MAMOT3112-13|Idaea eremica|COI-5P|KX861293
MAMOT3129-13|Idaea eremica|COI-5P|KX862242
MAMOT3741-13|Idaea eremica|COI-5P|KX861528
GWORM246-09|Idaea palaestinensis|COI-5P|HM393687
GWOUB683-19|Idaea palaestinensis|COI-5P
GWOR169-07|Idaea palaestinensis|COI-5P
GWORM247-09|Idaea palaestinensis|COI-5P|HM393688
GWORM243-09|Idaea palaestinensis|COI-5P|HM393686
GWORM239-09|Idaea palaestinensis|COI-5P|GU687151
GWORM236-09|Idaea palaestinensis|COI-5P|GU687148
GWORM235-09|Idaea palaestinensis|COI-5P|GU687155
GWOR180-07|Idaea palaestinensis|COI-5P
GWOR167-07|Idaea palaestinensis|COI-5P
GWORM233-09|Idaea palaestinensis|COI-5P|GU687153
GWORM241-09|Idaea palaestinensis|COI-5P|HM393685
GWOUB687-19|Idaea palaestinensis|COI-5P
GWORA1344-08|Idaea persidis|COI-5P
MAMOT3111-13|Idaea PK01|COI-5P|KX862148
GWORL755-09|Idaea epaphrodita|COI-5P|HM422575
GWORL869-09|Idaea obsoletaria|COI-5P|GU686633
GWORL931-09|Idaea obsoletaria|COI-5P|GU686576
GWORL868-09|Idaea obsoletaria|COI-5P|GU686640
GWORQ469-10|Idaea obsoletaria|COI-5P|HM903808
GWOSI547-10|Idaea obsoletaria|COI-5P|JN285251
GWORE1181-08|Idaea obsoletaria|COI-5P
GWORE1182-08|Idaea obsoletaria|COI-5P
PHLSA620-11|Idaea obsoletaria|COI-5P|KF807194
GWOTI800-12|Idaea obsoletaria|COI-5P|KF807851
LEATJ1071-15|Idaea obsoletaria|COI-5P
PHLAC570-10|Idaea obsoletaria|COI-5P|JF860138
GWORE622-08|Idaea elongaria|COI-5P|KF807408
GWOSP889-11|Idaea elongaria|COI-5P|KF807550
GWORP761-09|Idaea elongaria|COI-5P|HM394194
GWOTI816-12|Idaea elongaria|COI-5P|KF807650
GWOSI166-10|Idaea elongaria|COI-5P|KF807467
GWOSF934-10|Idaea elongaria|COI-5P|KF807373
GBLAA568-14|Idaea elongaria|COI-5P
GWOSO531-11|Idaea elongaria|COI-5P|KF807850
GWOSN362-11|Idaea palmata|COI-5P|KX040871
LEATD615-13|Idaea elongaria|COI-5P
GWOR221-07|Idaea elongaria|COI-5P
GWOR222-07|Idaea elongaria|COI-5P
GWORC1160-08|Idaea elongaria|COI-5P|KF807779
GWORC923-08|Idaea elongaria|COI-5P
GWORI431-09|Idaea fathmaria|COI-5P
GWORU510-10|Idaea carvalhoi|COI-5P|HM910647

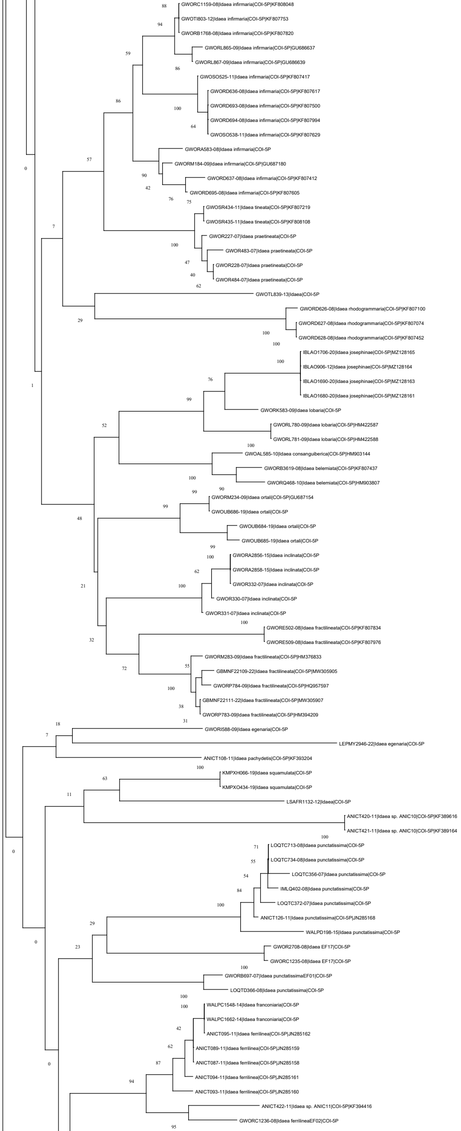
GWORC1159-08|Idaea infirmaria|COI-5P|KF808048
GWOTI803-12|Idaea infirmaria|COI-5P|KF807753
GWORB1768-08|Idaea infirmaria|COI-5P|KF807820
GWORL865-09|Idaea infirmaria|COI-5P|GU686637
GWORL867-09|Idaea infirmaria|COI-5P|GU686639
GWOSO525-11|Idaea infirmaria|COI-5P|KF807417
GWORD636-08|Idaea infirmaria|COI-5P|KF807617
GWORD693-08|Idaea infirmaria|COI-5P|KF807500
GWORD694-08|Idaea infirmaria|COI-5P|KF807994
GWOSO538-11|Idaea infirmaria|COI-5P|KF807629
GWORA583-08|Idaea infirmaria|COI-5P
GWORM184-09|Idaea infirmaria|COI-5P|GU687180
GWORD637-08|Idaea infirmaria|COI-5P|KF807412
GWORD695-08|Idaea infirmaria|COI-5P|KF807605
GWOSR434-11|Idaea tineata|COI-5P|KF807219
GWOSR435-11|Idaea tineata|COI-5P|KF808108
GWOR227-07|Idaea praetineata|COI-5P
GWOR483-07|Idaea praetineata|COI-5P
GWOR228-07|Idaea praetineata|COI-5P
GWOR484-07|Idaea praetineata|COI-5P
GWOTL839-13|Idaea|COI-5P
GWORD626-08|Idaea rhodogrammaria|COI-5P|KF807100
GWORD627-08|Idaea rhodogrammaria|COI-5P|KF807074
GWORD628-08|Idaea rhodogrammaria|COI-5P|KF807452
IBLAO1706-20|Idaea josephinae|COI-5P|MZ128165
IBLAO906-12|Idaea josephinae|COI-5P|MZ128164
IBLAO1690-20|Idaea josephinae|COI-5P|MZ128163
IBLAO1680-20|Idaea josephinae|COI-5P|MZ128161
GWORK583-09|Idaea lobaria|COI-5P
GWORL780-09|Idaea lobaria|COI-5P|HM422587
GWORL781-09|Idaea lobaria|COI-5P|HM422588
GWOAL585-10|Idaea consanguiberica|COI-5P|HM903144
GWORB3619-08|Idaea belemiata|COI-5P|KF807437
GWORQ468-10|Idaea belemiata|COI-5P|HM903807
GWORM234-09|Idaea ortali|COI-5P|GU687154
GWOUB686-19|Idaea ortali|COI-5P
GWOUB684-19|Idaea ortali|COI-5P
GWOUB685-19|Idaea ortali|COI-5P
GWORA2856-15|Idaea inclinata|COI-5P
GWORA2858-15|Idaea inclinata|COI-5P
GWOR332-07|Idaea inclinata|COI-5P
GWOR330-07|Idaea inclinata|COI-5P
GWOR331-07|Idaea inclinata|COI-5P
GWORE502-08|Idaea fractilineata|COI-5P|KF807834
GWORE509-08|Idaea fractilineata|COI-5P|KF807976
GWORM283-09|Idaea fractilineata|COI-5P|HM376833
GBMNF22109-22|Idaea fractilineata|COI-5P|MW305905
GWORP784-09|Idaea fractilineata|COI-5P|HQ957597
GBMNF22111-22|Idaea fractilineata|COI-5P|MW305907
GWORP783-09|Idaea fractilineata|COI-5P|HM394209
GWORI588-09|Idaea egenaria|COI-5P
LEPMY2946-22|Idaea egenaria|COI-5P
ANICT108-11|Idaea pachydetis|COI-5P|KF393204
KMPXH066-19|Idaea squamulata|COI-5P
KMPXO434-19|Idaea squamulata|COI-5P
LSAFR1132-12|Idaea|COI-5P
ANICT420-11|Idaea sp. ANIC10|COI-5P|KF389616
ANICT421-11|Idaea sp. ANIC10|COI-5P|KF389164
LOQTC713-08|Idaea punctatissima|COI-5P
LOQTC734-08|Idaea punctatissima|COI-5P
LOQTC356-07|Idaea punctatissima|COI-5P
IMLQ402-08|Idaea punctatissima|COI-5P
LOQTC372-07|Idaea punctatissima|COI-5P
ANICT126-11|Idaea punctatissima|COI-5P|JN285168
WALPD198-15|Idaea punctatissima|COI-5P
GWOR2708-08|Idaea EF17|COI-5P
GWORC1235-08|Idaea EF17|COI-5P
GWORB697-07|Idaea punctatissimaEF01|COI-5P
LOQTD366-08|Idaea punctatissima|COI-5P
WALPC1548-14|Idaea franconiaria|COI-5P
WALPC1662-14|Idaea franconiaria|COI-5P
ANICT095-11|Idaea ferrilinea|COI-5P|JN285162
ANICT089-11|Idaea ferrilinea|COI-5P|JN285159
ANICT087-11|Idaea ferrilinea|COI-5P|JN285158
ANICT094-11|Idaea ferrilinea|COI-5P|JN285161
ANICT093-11|Idaea ferrilinea|COI-5P|JN285160
ANICT422-11|Idaea sp. ANIC11|COI-5P|KF394416
GWORC1236-08|Idaea ferrilineaEF02|COI-5P

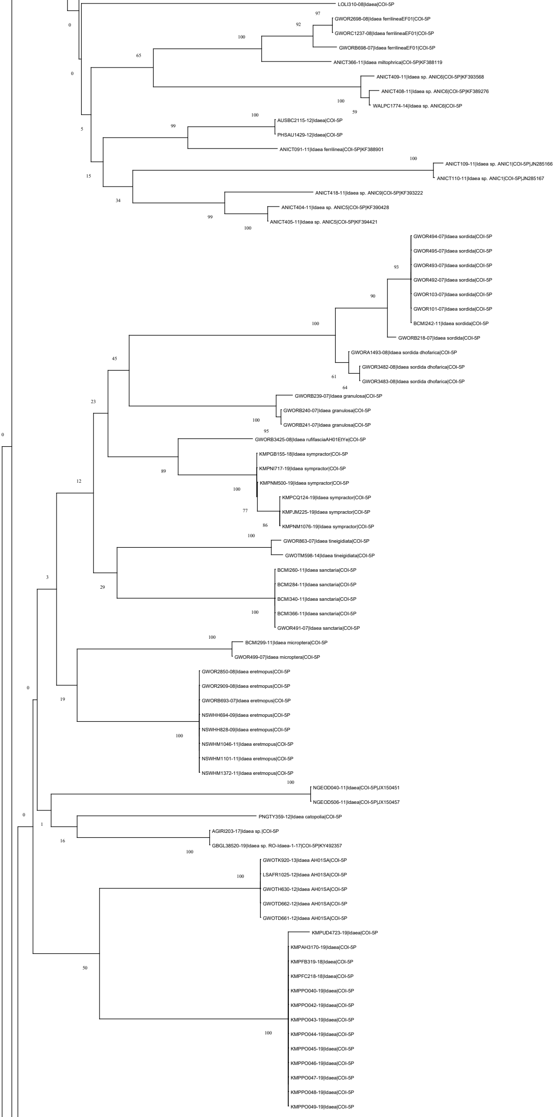

LOLI310-08|Idaea|COI-5P
0
97
GWOR2698-08|Idaea ferrilineaEF01|COI-5P
92
GWORC1237-08|Idaea ferrilineaEF01|COI-5P
100
GWORB698-07|Idaea ferrilineaEF01|COI-5P
65
ANICT366-11|Idaea miltophrica|COI-5P|KF388119
0
ANICT409-11|Idaea sp. ANIC6|COI-5P|KF393568
ANICT408-11|Idaea sp. ANIC6|COI-5P|KF389276
100
WALPC1774-14|Idaea sp. ANIC6|COI-5P
59
100
AUSBC2115-12|Idaea|COI-5P
99
5
PHSAU1429-12|Idaea|COI-5P
ANICT091-11|Idaea ferrilinea|COI-5P|KF388901
100
ANICT109-11|Idaea sp. ANIC1|COI-5P|JN285166
15
ANICT110-11|Idaea sp. ANIC1|COI-5P|JN285167
ANICT418-11|Idaea sp. ANIC9|COI-5P|KF393222
34
ANICT404-11|Idaea sp. ANIC5|COI-5P|KF390428
99
ANICT405-11|Idaea sp. ANIC5|COI-5P|KF394421
100
GWOR494-07|Idaea sordida|COI-5P
GWOR495-07|Idaea sordida|COI-5P
93
GWOR493-07|Idaea sordida|COI-5P
GWOR492-07|Idaea sordida|COI-5P
90
GWOR103-07|Idaea sordida|COI-5P
GWOR101-07|Idaea sordida|COI-5P
100
BCMI242-11|Idaea sordida|COI-5P
GWORB218-07|Idaea sordida|COI-5P
GWORA1493-08|Idaea sordida dhofarica|COI-5P
45
GWOR3482-08|Idaea sordida dhofarica|COI-5P
61
GWOR3483-08|Idaea sordida dhofarica|COI-5P
64
23
GWORB239-07|Idaea granulosa|COI-5P
GWORB240-07|Idaea granulosa|COI-5P
100
GWORB241-07|Idaea granulosa|COI-5P
95
0
GWORB3425-08|Idaea rufifasciaAH01EtYe|COI-5P
KMPGB155-18|Idaea sympractor|COI-5P
KMPNI717-19|Idaea sympractor|COI-5P
89
12
KMPNM500-19|Idaea sympractor|COI-5P
100
KMPCQ124-19|Idaea sympractor|COI-5P
KMPJM225-19|Idaea sympractor|COI-5P
77
86
KMPNM1076-19|Idaea sympractor|COI-5P
100
GWOR863-07|Idaea tineigidiata|COI-5P
GWOTM598-14|Idaea tineigidiata|COI-5P
BCMI260-11|Idaea sanctaria|COI-5P
3
BCMI284-11|Idaea sanctaria|COI-5P
29
BCMI340-11|Idaea sanctaria|COI-5P
100
BCMI366-11|Idaea sanctaria|COI-5P
GWOR491-07|Idaea sanctaria|COI-5P
100
BCMI299-11|Idaea microptera|COI-5P
GWOR499-07|Idaea microptera|COI-5P
GWOR2850-08|Idaea eretmopus|COI-5P
0
GWOR2909-08|Idaea eretmopus|COI-5P
19
GWORB693-07|Idaea eretmopus|COI-5P
NSWHH694-09|Idaea eretmopus|COI-5P
NSWHH828-09|Idaea eretmopus|COI-5P
100
NSWHM1046-11|Idaea eretmopus|COI-5P
NSWHM1101-11|Idaea eretmopus|COI-5P
NSWHM1372-11|Idaea eretmopus|COI-5P
100
NGEOD040-11|Idaea|COI-5P|JX150451
NGEOD506-11|Idaea|COI-5P|JX150457
0
PNGTY359-12|Idaea catopolia|COI-5P
1
AGIRI203-17|Idaea sp.|COI-5P
16
GBGL38520-19|Idaea sp. RO-Idaea-1-17|COI-5P|KY492357
100
GWOTK920-13|Idaea AH01SA|COI-5P
100
LSAFR1025-12|Idaea AH01SA|COI-5P
GWOTH630-12|Idaea AH01SA|COI-5P
GWOTD662-12|Idaea AH01SA|COI-5P
GWOTD661-12|Idaea AH01SA|COI-5P
KMPUD4723-19|Idaea|COI-5P
KMPAH3170-19|Idaea|COI-5P
KMPFB319-18|Idaea|COI-5P
50
KMPFC218-18|Idaea|COI-5P
KMPPO040-19|Idaea|COI-5P
KMPPO042-19|Idaea|COI-5P
KMPPO043-19|Idaea|COI-5P
100
KMPPO044-19|Idaea|COI-5P
KMPPO045-19|Idaea|COI-5P
KMPPO046-19|Idaea|COI-5P
KMPPO047-19|Idaea|COI-5P
KMPPO048-19|Idaea|COI-5P
KMPPO049-19|Idaea|COI-5P

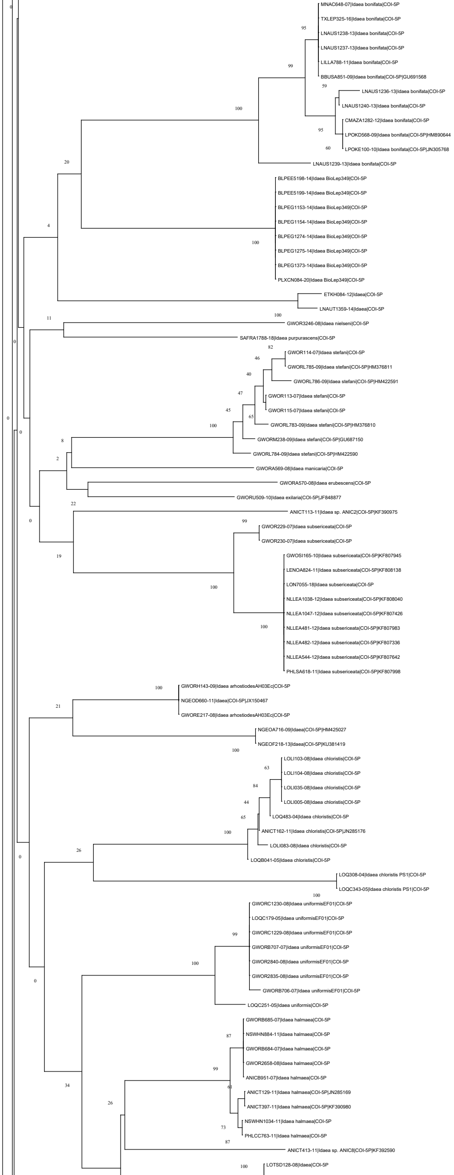

MNAC648-07|Idaea bonifata|COI-5P
TXLEP325-16|Idaea bonifata|COI-5P
LNAUS1238-13|Idaea bonifata|COI-5P
LNAUS1237-13|Idaea bonifata|COI-5P
LILLA788-11|Idaea bonifata|COI-5P
BBUSA851-09|Idaea bonifata|COI-5P|GU691568
LNAUS1236-13|Idaea bonifata|COI-5P
LNAUS1240-13|Idaea bonifata|COI-5P
CMAZA1282-12|Idaea bonifata|COI-5P
LPOKD568-09|Idaea bonifata|COI-5P|HM890644
LPOKE100-10|Idaea bonifata|COI-5P|JN305768
LNAUS1239-13|Idaea bonifata|COI-5P
BLPEE5198-14|Idaea BioLep349|COI-5P
BLPEE5199-14|Idaea BioLep349|COI-5P
BLPEG1153-14|Idaea BioLep349|COI-5P
BLPEG1154-14|Idaea BioLep349|COI-5P
BLPEG1274-14|Idaea BioLep349|COI-5P
BLPEG1275-14|Idaea BioLep349|COI-5P
BLPEG1373-14|Idaea BioLep349|COI-5P
PLXCN084-20|Idaea BioLep349|COI-5P
ETKH084-12|Idaea|COI-5P
LNAUT1359-14|Idaea|COI-5P
GWOR3246-08|Idaea nielseni|COI-5P
SAFRA1788-18|Idaea purpurascens|COI-5P
GWOR114-07|Idaea stefani|COI-5P
GWORL785-09|Idaea stefani|COI-5P|HM376811
GWORL786-09|Idaea stefani|COI-5P|HM422591
GWOR113-07|Idaea stefani|COI-5P
GWOR115-07|Idaea stefani|COI-5P
GWORL783-09|Idaea stefani|COI-5P|HM376810
GWORM238-09|Idaea stefani|COI-5P|GU687150
GWORL784-09|Idaea stefani|COI-5P|HM422590
GWORA569-08|Idaea manicaria|COI-5P
GWORA570-08|Idaea erubescens|COI-5P
GWORU509-10|Idaea exilaria|COI-5P|JF848877
ANICT113-11|Idaea sp. ANIC2|COI-5P|KF390975
GWOR229-07|Idaea subsericeata|COI-5P
GWOR230-07|Idaea subsericeata|COI-5P
GWOSI165-10|Idaea subsericeata|COI-5P|KF807945
LENOA824-11|Idaea subsericeata|COI-5P|KF808138
LON7055-18|Idaea subsericeata|COI-5P
NLLEA1038-12|Idaea subsericeata|COI-5P|KF808040
NLLEA1047-12|Idaea subsericeata|COI-5P|KF807426
NLLEA481-12|Idaea subsericeata|COI-5P|KF807983
NLLEA482-12|Idaea subsericeata|COI-5P|KF807336
NLLEA544-12|Idaea subsericeata|COI-5P|KF807642
PHLSA618-11|Idaea subsericeata|COI-5P|KF807998
GWORH143-09|Idaea arhostiodesAH03Ec|COI-5P
NGEOD660-11|Idaea|COI-5P|JX150467
GWORE217-08|Idaea arhostiodesAH03Ec|COI-5P
NGEOA716-09|Idaea|COI-5P|HM425027
NGEOF218-13|Idaea|COI-5P|KU381419
LOLI103-08|Idaea chloristis|COI-5P
LOLI104-08|Idaea chloristis|COI-5P
LOLI035-08|Idaea chloristis|COI-5P
LOLI005-08|Idaea chloristis|COI-5P
LOQ483-04|Idaea chloristis|COI-5P
ANICT162-11|Idaea chloristis|COI-5P|JN285176
LOLI083-08|Idaea chloristis|COI-5P
LOQB041-05|Idaea chloristis|COI-5P
LOQ308-04|Idaea chloristis PS1|COI-5P
LOQC343-05|Idaea chloristis PS1|COI-5P
GWORC1230-08|Idaea uniformisEF01|COI-5P
LOQC179-05|Idaea uniformisEF01|COI-5P
GWORC1229-08|Idaea uniformisEF01|COI-5P
GWORB707-07|Idaea uniformisEF01|COI-5P
GWOR2840-08|Idaea uniformisEF01|COI-5P
GWOR2835-08|Idaea uniformisEF01|COI-5P
GWORB706-07|Idaea uniformisEF01|COI-5P
LOQC251-05|Idaea uniformis|COI-5P
GWORB685-07|Idaea halmaea|COI-5P
NSWHN884-11|Idaea halmaea|COI-5P
GWORB684-07|Idaea halmaea|COI-5P
GWOR2658-08|Idaea halmaea|COI-5P
ANICB951-07|Idaea halmaea|COI-5P
ANICT129-11|Idaea halmaea|COI-5P|JN285169
ANICT397-11|Idaea halmaea|COI-5P|KF390980
NSWHN1034-11|Idaea halmaea|COI-5P
PHLCC763-11|Idaea halmaea|COI-5P
ANICT413-11|Idaea sp. ANIC8|COI-5P|KF392590
LOTSD128-08|Idaea|COI-5P

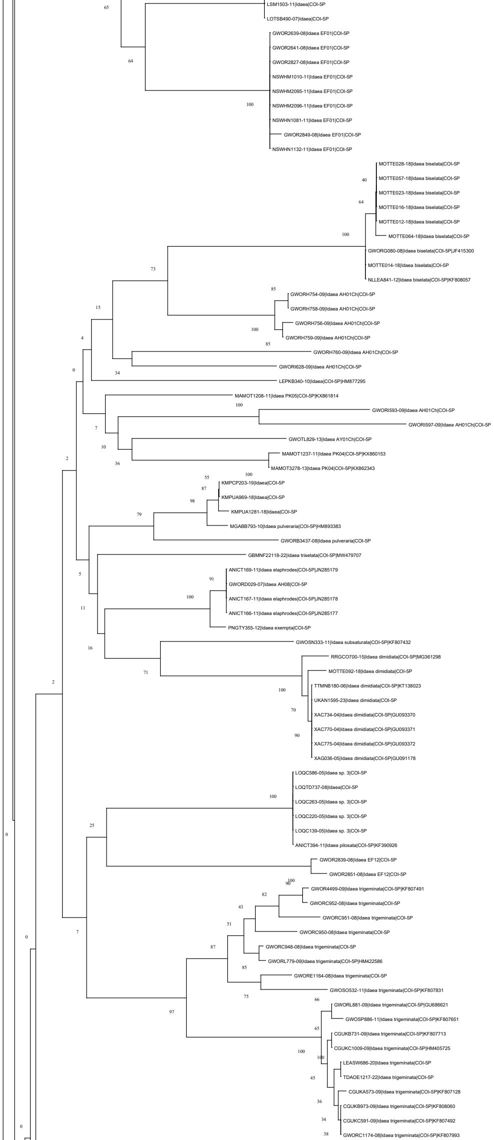

LSM1503-11|Idaea|COI-5P
LOTSB490-07|Idaea|COI-5P
GWOR2639-08|Idaea EF01|COI-5P
GWOR2641-08|Idaea EF01|COI-5P
GWOR2827-08|Idaea EF01|COI-5P
NSWHM1010-11|Idaea EF01|COI-5P
NSWHM2095-11|Idaea EF01|COI-5P
NSWHM2096-11|Idaea EF01|COI-5P
NSWHN1081-11|Idaea EF01|COI-5P
GWOR2849-08|Idaea EF01|COI-5P
NSWHN1132-11|Idaea EF01|COI-5P
MOTTE028-18|Idaea biselata|COI-5P
MOTTE057-18|Idaea biselata|COI-5P
MOTTE023-18|Idaea biselata|COI-5P
MOTTE016-18|Idaea biselata|COI-5P
MOTTE012-18|Idaea biselata|COI-5P
MOTTE064-18|Idaea biselata|COI-5P
GWORG080-08|Idaea biselata|COI-5P|JF415300
MOTTE014-18|Idaea biselata|COI-5P
NLLEA841-12|Idaea biselata|COI-5P|KF808057
GWORH754-09|Idaea AH01Ch|COI-5P
GWORH758-09|Idaea AH01Ch|COI-5P
GWORH756-09|Idaea AH01Ch|COI-5P
GWORH759-09|Idaea AH01Ch|COI-5P
GWORH760-09|Idaea AH01Ch|COI-5P
GWORI628-09|Idaea AH01Ch|COI-5P
LEPKB340-10|Idaea|COI-5P|HM877295
MAMOT1208-11|Idaea PK05|COI-5P|KX861814
GWORI593-09|Idaea AH01Ch|COI-5P
GWORI597-09|Idaea AH01Ch|COI-5P
GWOTL829-13|Idaea AY01Ch|COI-5P
MAMOT1237-11|Idaea PK04|COI-5P|KX860153
MAMOT3278-13|Idaea PK04|COI-5P|KX862343
KMPCP203-19|Idaea|COI-5P
KMPUA969-18|Idaea|COI-5P
KMPUA1281-18|Idaea|COI-5P
MGABB793-10|Idaea pulveraria|COI-5P|HM893383
GWORB3437-08|Idaea pulveraria|COI-5P
GBMNF22118-22|Idaea trisetata|COI-5P|MW479707
ANICT169-11|Idaea elaphrodes|COI-5P|JN285179
GWORD029-07|Idaea AH08|COI-5P
ANICT167-11|Idaea elaphrodes|COI-5P|JN285178
ANICT166-11|Idaea elaphrodes|COI-5P|JN285177
PNGTY355-12|Idaea exempta|COI-5P
GWOSN333-11|Idaea subsaturata|COI-5P|KF807432
RRGCO700-15|Idaea dimidiata|COI-5P|MG361298
MOTTE092-18|Idaea dimidiata|COI-5P
TTMNB180-06|Idaea dimidiata|COI-5P|KT138023
UKAN1595-23|Idaea dimidiata|COI-5P
XAC734-04|Idaea dimidiata|COI-5P|GU093370
XAC770-04|Idaea dimidiata|COI-5P|GU093371
XAC775-04|Idaea dimidiata|COI-5P|GU093372
XAG036-05|Idaea dimidiata|COI-5P|GU091178
LOQC586-05|Idaea sp. 3|COI-5P
LOQTD737-08|Idaea|COI-5P
LOQC263-05|Idaea sp. 3|COI-5P
LOQC220-05|Idaea sp. 3|COI-5P
LOQC139-05|Idaea sp. 3|COI-5P
ANICT394-11|Idaea pilosata|COI-5P|KF390926
GWOR2839-08|Idaea EF12|COI-5P
GWOR2851-08|Idaea EF12|COI-5P
GWOR4499-09|Idaea trigeminata|COI-5P|KF807491
GWORC952-08|Idaea trigeminata|COI-5P
GWORC951-08|Idaea trigeminata|COI-5P
GWORC950-08|Idaea trigeminata|COI-5P
GWORC948-08|Idaea trigeminata|COI-5P
GWORL779-09|Idaea trigeminata|COI-5P|HM422586
GWORE1164-08|Idaea trigeminata|COI-5P
GWOSO532-11|Idaea trigeminata|COI-5P|KF807831
GWORL881-09|Idaea trigeminata|COI-5P|GU686621
GWOSP886-11|Idaea trigeminata|COI-5P|KF807651
CGUKB731-09|Idaea trigeminata|COI-5P|KF807713
CGUKC1009-09|Idaea trigeminata|COI-5P|HM405725
LEASW686-20|Idaea trigeminata|COI-5P
TDAOE1217-22|Idaea trigeminata|COI-5P
CGUKA573-09|Idaea trigeminata|COI-5P|KF807128
CGUKB973-09|Idaea trigeminata|COI-5P|KF808060
CGUKC591-09|Idaea trigeminata|COI-5P|KF807492
GWORC1174-08|Idaea trigeminata|COI-5P|KF807993

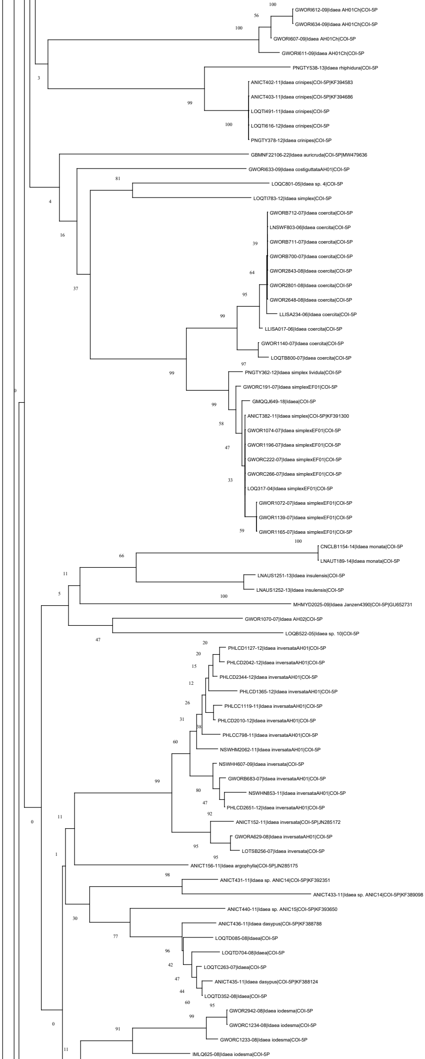
GWORI612-09|Idaea AH01Ch|COI-5P
GWORI634-09|Idaea AH01Ch|COI-5P
GWORI607-09|Idaea AH01Ch|COI-5P
GWORI611-09|Idaea AH01Ch|COI-5P
PNGTY538-13|Idaea rhiphidura|COI-5P
ANICT402-11|Idaea crinipes|COI-5P|KF394583
ANICT403-11|Idaea crinipes|COI-5P|KF394686
LOQTI491-11|Idaea crinipes|COI-5P
LOQTI616-12|Idaea crinipes|COI-5P
PNGTY378-12|Idaea crinipes|COI-5P
GBMNF22106-22|Idaea auricruda|COI-5P|MW479636
GWORI633-09|Idaea costiguttataAH01|COI-5P
LOQC801-05|Idaea sp. 4|COI-5P
LOQTI783-12|Idaea simplex|COI-5P
GWORB712-07|Idaea coercita|COI-5P
LNSWF803-06|Idaea coercita|COI-5P
GWORB711-07|Idaea coercita|COI-5P
GWORB700-07|Idaea coercita|COI-5P
GWOR2843-08|Idaea coercita|COI-5P
GWOR2801-08|Idaea coercita|COI-5P
GWOR2648-08|Idaea coercita|COI-5P
LLISA234-06|Idaea coercita|COI-5P
LLISA017-06|Idaea coercita|COI-5P
GWOR1140-07|Idaea coercita|COI-5P
LOQTB800-07|Idaea coercita|COI-5P
PNGTY362-12|Idaea simplex lividula|COI-5P
GWORC191-07|Idaea simplexEF01|COI-5P
GMQQJ649-18|Idaea|COI-5P
ANICT382-11|Idaea simplex|COI-5P|KF391300
GWOR1074-07|Idaea simplexEF01|COI-5P
GWOR1196-07|Idaea simplexEF01|COI-5P
GWORC222-07|Idaea simplexEF01|COI-5P
GWORC266-07|Idaea simplexEF01|COI-5P
LOQ317-04|Idaea simplexEF01|COI-5P
GWOR1072-07|Idaea simplexEF01|COI-5P
GWOR1139-07|Idaea simplexEF01|COI-5P
GWOR1165-07|Idaea simplexEF01|COI-5P
CNCLB1154-14|Idaea monata|COI-5P
LNAUT189-14|Idaea monata|COI-5P
LNAUS1251-13|Idaea insulensis|COI-5P
LNAUS1252-13|Idaea insulensis|COI-5P
MHMYD2025-09|Idaea Janzen4390|COI-5P|GU652731
GWOR1070-07|Idaea AH02|COI-5P
LOQB522-05|Idaea sp. 10|COI-5P
PHLCD1127-12|Idaea inversataAH01|COI-5P
PHLCD2042-12|Idaea inversataAH01|COI-5P
PHLCD2344-12|Idaea inversataAH01|COI-5P
PHLCD1365-12|Idaea inversataAH01|COI-5P
PHLCC1119-11|Idaea inversataAH01|COI-5P
PHLCD2010-12|Idaea inversataAH01|COI-5P
PHLCC798-11|Idaea inversataAH01|COI-5P
NSWHM2062-11|Idaea inversataAH01|COI-5P
NSWHH607-09|Idaea inversata|COI-5P
GWORB683-07|Idaea inversataAH01|COI-5P
NSWHN853-11|Idaea inversataAH01|COI-5P
PHLCD2651-12|Idaea inversataAH01|COI-5P
ANICT152-11|Idaea inversata|COI-5P|JN285172
GWORA629-08|Idaea inversataAH01|COI-5P
LOTSB256-07|Idaea inversata|COI-5P
ANICT156-11|Idaea argophylla|COI-5P|JN285175
ANICT431-11|Idaea sp. ANIC14|COI-5P|KF392351
ANICT433-11|Idaea sp. ANIC14|COI-5P|KF389098
ANICT440-11|Idaea sp. ANIC15|COI-5P|KF393650
ANICT436-11|Idaea dasypus|COI-5P|KF388788
LOQTD085-08|Idaea|COI-5P
LOQTD704-08|Idaea|COI-5P
LOQTC263-07|Idaea|COI-5P
ANICT435-11|Idaea dasypus|COI-5P|KF388124
LOQTD352-08|Idaea|COI-5P
GWOR2942-08|Idaea iodesma|COI-5P
GWORC1234-08|Idaea iodesma|COI-5P
GWORC1233-08|Idaea iodesma|COI-5P
IMLQ625-08|Idaea iodesma|COI-5P

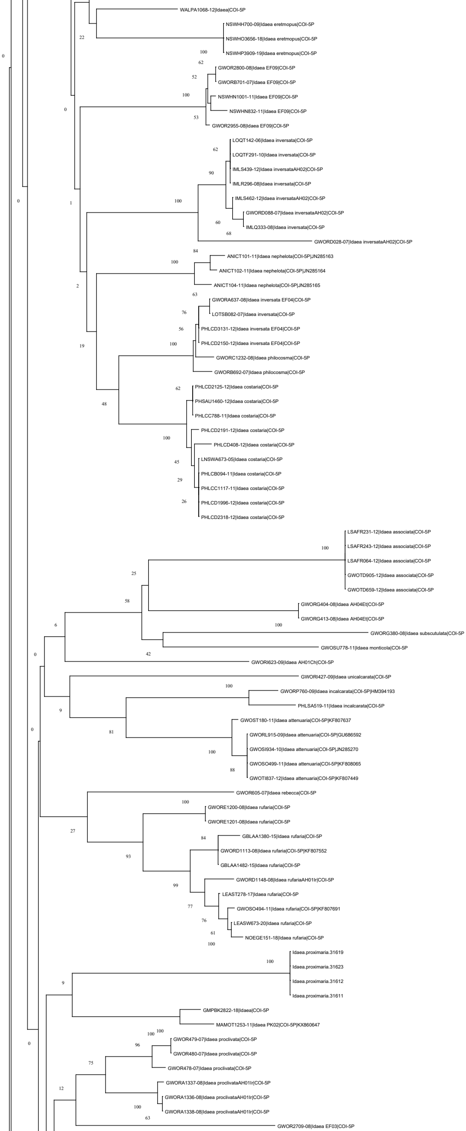

WALPA1068-12|Idaea|COI-5P
NSWHH700-09|Idaea eretmopus|COI-5P
NSWHO3656-18|Idaea eretmopus|COI-5P
NSWHP3909-19|Idaea eretmopus|COI-5P
GWOR2800-08|Idaea EF09|COI-5P
GWORB701-07|Idaea EF09|COI-5P
NSWHN1001-11|Idaea EF09|COI-5P
NSWHN832-11|Idaea EF09|COI-5P
GWOR2955-08|Idaea EF09|COI-5P
LOQT142-06|Idaea inversata|COI-5P
LOQTF291-10|Idaea inversata|COI-5P
IMLS439-12|Idaea inversataAH02|COI-5P
IMLR296-08|Idaea inversata|COI-5P
IMLS462-12|Idaea inversataAH02|COI-5P
GWORD088-07|Idaea inversataAH02|COI-5P
IMLQ333-08|Idaea inversata|COI-5P
GWORD028-07|Idaea inversataAH02|COI-5P
ANICT101-11|Idaea nephelota|COI-5P|JN285163
ANICT102-11|Idaea nephelota|COI-5P|JN285164
ANICT104-11|Idaea nephelota|COI-5P|JN285165
GWORA637-08|Idaea inversata EF04|COI-5P
LOTSB082-07|Idaea inversata|COI-5P
PHLCD3131-12|Idaea inversata EF04|COI-5P
PHLCD2150-12|Idaea inversata EF04|COI-5P
GWORC1232-08|Idaea philocosma|COI-5P
GWORB692-07|Idaea philocosma|COI-5P
PHLCD2125-12|Idaea costaria|COI-5P
PHSAU1460-12|Idaea costaria|COI-5P
PHLCC788-11|Idaea costaria|COI-5P
PHLCD2191-12|Idaea costaria|COI-5P
PHLCD408-12|Idaea costaria|COI-5P
LNSWA673-05|Idaea costaria|COI-5P
PHLCB094-11|Idaea costaria|COI-5P
PHLCC1117-11|Idaea costaria|COI-5P
PHLCD1996-12|Idaea costaria|COI-5P
PHLCD2318-12|Idaea costaria|COI-5P
LSAFR231-12|Idaea associata|COI-5P
LSAFR243-12|Idaea associata|COI-5P
LSAFR064-12|Idaea associata|COI-5P
GWOTD905-12|Idaea associata|COI-5P
GWOTD659-12|Idaea associata|COI-5P
GWORG404-08|Idaea AH04Et|COI-5P
GWORG413-08|Idaea AH04Et|COI-5P
GWORG380-08|Idaea subscutulata|COI-5P
GWOSU778-11|Idaea monticola|COI-5P
GWORI623-09|Idaea AH01Ch|COI-5P
GWORI427-09|Idaea unicalcarata|COI-5P
GWORP760-09|Idaea incalcarata|COI-5P|HM394193
PHLSA519-11|Idaea incalcarata|COI-5P
GWOST180-11|Idaea attenuaria|COI-5P|KF807637
GWORL915-09|Idaea attenuaria|COI-5P|GU686592
GWOSI934-10|Idaea attenuaria|COI-5P|JN285270
GWOSO499-11|Idaea attenuaria|COI-5P|KF808065
GWOTI837-12|Idaea attenuaria|COI-5P|KF807449
GWOR605-07|Idaea rebecca|COI-5P
GWORE1200-08|Idaea rufaria|COI-5P
GWORE1201-08|Idaea rufaria|COI-5P
GBLAA1380-15|Idaea rufaria|COI-5P
GWORD1113-08|Idaea rufaria|COI-5P|KF807552
GBLAA1482-15|Idaea rufaria|COI-5P
GWORD1148-08|Idaea rufariaAH01Ir|COI-5P
LEAST278-17|Idaea rufaria|COI-5P
GWOSO494-11|Idaea rufaria|COI-5P|KF807691
LEASW673-20|Idaea rufaria|COI-5P
NOEGE151-18|Idaea rufaria|COI-5P
Idaea.proximaria.31619
Idaea.proximaria.31623
Idaea.proximaria.31612
Idaea.proximaria.31611
GMPBK2822-18|Idaea|COI-5P
MAMOT1253-11|Idaea PK02|COI-5P|KX860647
GWOR479-07|Idaea proclivata|COI-5P
GWOR480-07|Idaea proclivata|COI-5P
GWOR478-07|Idaea proclivata|COI-5P
GWORA1337-08|Idaea proclivataAH01Ir|COI-5P
GWORA1336-08|Idaea proclivataAH01Ir|COI-5P
GWORA1338-08|Idaea proclivataAH01Ir|COI-5P
GWOR2709-08|Idaea EF03|COI-5P

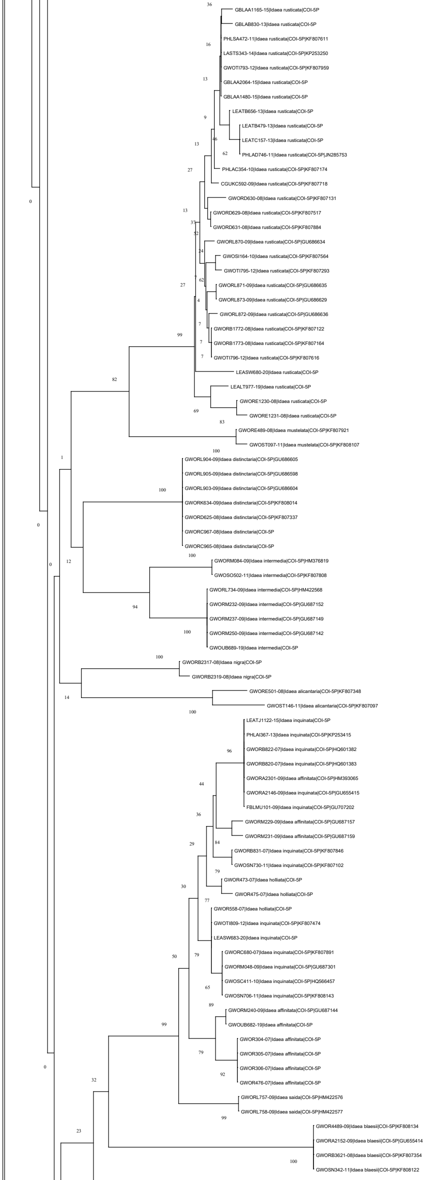
GBLAA1165-15|Idaea rusticata|COI-5P
GBLAB830-13|Idaea rusticata|COI-5P
PHLSA472-11|Idaea rusticata|COI-5P|KF807611
LASTS343-14|Idaea rusticata|COI-5P|KP253250
GWOTI793-12|Idaea rusticata|COI-5P|KF807959
GBLAA2064-15|Idaea rusticata|COI-5P
GBLAA1480-15|Idaea rusticata|COI-5P
LEATB656-13|Idaea rusticata|COI-5P
LEATB479-13|Idaea rusticata|COI-5P
LEATC157-13|Idaea rusticata|COI-5P
PHLAD746-11|Idaea rusticata|COI-5P|JN285753
PHLAC354-10|Idaea rusticata|COI-5P|KF807174
CGUKC592-09|Idaea rusticata|COI-5P|KF807718
GWORD630-08|Idaea rusticata|COI-5P|KF807131
GWORD629-08|Idaea rusticata|COI-5P|KF807517
GWORD631-08|Idaea rusticata|COI-5P|KF807884
GWORL870-09|Idaea rusticata|COI-5P|GU686634
GWOSI164-10|Idaea rusticata|COI-5P|KF807564
GWOTI795-12|Idaea rusticata|COI-5P|KF807293
GWORL871-09|Idaea rusticata|COI-5P|GU686635
GWORL873-09|Idaea rusticata|COI-5P|GU686629
GWORL872-09|Idaea rusticata|COI-5P|GU686636
GWORB1772-08|Idaea rusticata|COI-5P|KF807122
GWORB1773-08|Idaea rusticata|COI-5P|KF807164
GWOTI796-12|Idaea rusticata|COI-5P|KF807616
LEASW680-20|Idaea rusticata|COI-5P
LEALT977-19|Idaea rusticata|COI-5P
GWORE1230-08|Idaea rusticata|COI-5P
GWORE1231-08|Idaea rusticata|COI-5P
GWORE489-08|Idaea mustelata|COI-5P|KF807921
GWOST097-11|Idaea mustelata|COI-5P|KF808107
GWORL904-09|Idaea distinctaria|COI-5P|GU686605
GWORL905-09|Idaea distinctaria|COI-5P|GU686598
GWORL903-09|Idaea distinctaria|COI-5P|GU686604
GWORK634-09|Idaea distinctaria|COI-5P|KF808014
GWORD625-08|Idaea distinctaria|COI-5P|KF807337
GWORC967-08|Idaea distinctaria|COI-5P
GWORC965-08|Idaea distinctaria|COI-5P
GWORM084-09|Idaea intermedia|COI-5P|HM376819
GWOSO502-11|Idaea intermedia|COI-5P|KF807808
GWORL734-09|Idaea intermedia|COI-5P|HM422568
GWORM232-09|Idaea intermedia|COI-5P|GU687152
GWORM237-09|Idaea intermedia|COI-5P|GU687149
GWORM250-09|Idaea intermedia|COI-5P|GU687142
GWOUB689-19|Idaea intermedia|COI-5P
GWORB2317-08|Idaea nigra|COI-5P
GWORB2319-08|Idaea nigra|COI-5P
GWORE501-08|Idaea alicantaria|COI-5P|KF807348
GWOST146-11|Idaea alicantaria|COI-5P|KF807097
LEATJ1122-15|Idaea inquinata|COI-5P
PHLAI367-13|Idaea inquinata|COI-5P|KP253415
GWORB822-07|Idaea inquinata|COI-5P|HQ601382
GWORB820-07|Idaea inquinata|COI-5P|HQ601383
GWORA2301-09|Idaea affinitata|COI-5P|HM393065
GWORA2146-09|Idaea inquinata|COI-5P|GU655415
FBLMU101-09|Idaea inquinata|COI-5P|GU707202
GWORM229-09|Idaea affinitata|COI-5P|GU687157
GWORM231-09|Idaea affinitata|COI-5P|GU687159
GWORB831-07|Idaea inquinata|COI-5P|KF807846
GWOSN730-11|Idaea inquinata|COI-5P|KF807102
GWOR473-07|Idaea holliata|COI-5P
GWOR475-07|Idaea holliata|COI-5P
GWOR558-07|Idaea holliata|COI-5P
GWOTI809-12|Idaea inquinata|COI-5P|KF807474
LEASW683-20|Idaea inquinata|COI-5P
GWORC680-07|Idaea inquinata|COI-5P|KF807891
GWORM048-09|Idaea inquinata|COI-5P|GU687301
GWOSC411-10|Idaea inquinata|COI-5P|HQ566457
GWOSN706-11|Idaea inquinata|COI-5P|KF808143
GWORM240-09|Idaea affinitata|COI-5P|GU687144
GWOUB682-19|Idaea affinitata|COI-5P
GWOR304-07|Idaea affinitata|COI-5P
GWOR305-07|Idaea affinitata|COI-5P
GWOR306-07|Idaea affinitata|COI-5P
GWOR476-07|Idaea affinitata|COI-5P
GWORL757-09|Idaea saida|COI-5P|HM422576
GWORL758-09|Idaea saida|COI-5P|HM422577
GWOR4489-09|Idaea blaesii|COI-5P|KF808134
GWORA2152-09|Idaea blaesii|COI-5P|GU655414
GWORB3621-08|Idaea blaesii|COI-5P|KF807354
GWOSN342-11|Idaea blaesii|COI-5P|KF808122

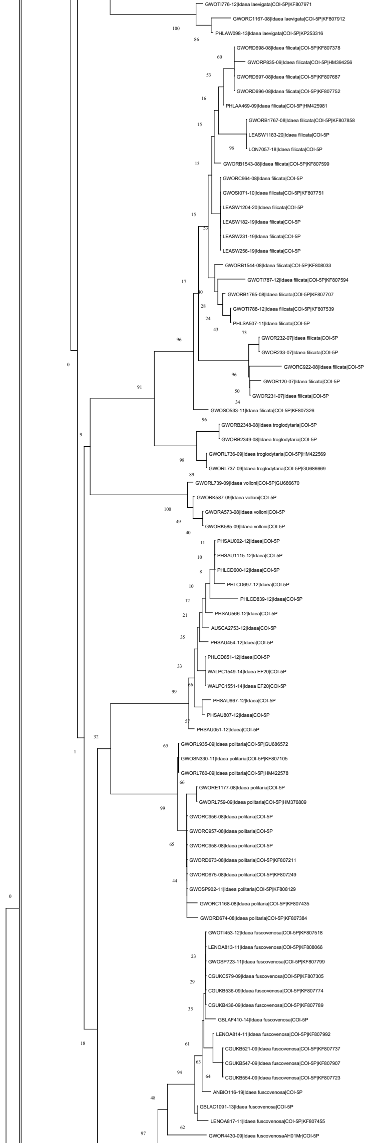
GWOTI776-12|Idaea laevigata|COI-5P|KF807971
GWORC1167-08|Idaea laevigata|COI-5P|KF807912
PHLAW098-13|Idaea laevigata|COI-5P|KP253316
GWORD698-08|Idaea filicata|COI-5P|KF807378
GWORP835-09|Idaea filicata|COI-5P|HM394256
GWORD697-08|Idaea filicata|COI-5P|KF807687
GWORD696-08|Idaea filicata|COI-5P|KF807752
PHLAA469-09|Idaea filicata|COI-5P|HM425981
GWORB1767-08|Idaea filicata|COI-5P|KF807858
LEASW1183-20|Idaea filicata|COI-5P
LON7057-18|Idaea filicata|COI-5P
GWORB1543-08|Idaea filicata|COI-5P|KF807599
GWORC964-08|Idaea filicata|COI-5P
GWOSI071-10|Idaea filicata|COI-5P|KF807751
LEASW1204-20|Idaea filicata|COI-5P
LEASW182-19|Idaea filicata|COI-5P
LEASW231-19|Idaea filicata|COI-5P
LEASW256-19|Idaea filicata|COI-5P
GWORB1544-08|Idaea filicata|COI-5P|KF808033
GWOTI787-12|Idaea filicata|COI-5P|KF807594
GWORB1765-08|Idaea filicata|COI-5P|KF807707
GWOTI788-12|Idaea filicata|COI-5P|KF807539
PHLSA507-11|Idaea filicata|COI-5P
GWOR232-07|Idaea filicata|COI-5P
GWOR233-07|Idaea filicata|COI-5P
GWORC922-08|Idaea filicata|COI-5P
GWOR120-07|Idaea filicata|COI-5P
GWOR231-07|Idaea filicata|COI-5P
GWOSO533-11|Idaea filicata|COI-5P|KF807326
GWORB2348-08|Idaea troglodytaria|COI-5P
GWORB2349-08|Idaea troglodytaria|COI-5P
GWORL736-09|Idaea troglodytaria|COI-5P|HM422569
GWORL737-09|Idaea troglodytaria|COI-5P|GU686669
GWORL739-09|Idaea volloni|COI-5P|GU686670
GWORK587-09|Idaea volloni|COI-5P
GWORA573-08|Idaea volloni|COI-5P
GWORK585-09|Idaea volloni|COI-5P
PHSAU002-12|Idaea|COI-5P
PHSAU1115-12|Idaea|COI-5P
PHLCD600-12|Idaea|COI-5P
PHLCD697-12|Idaea|COI-5P
PHLCD839-12|Idaea|COI-5P
PHSAU566-12|Idaea|COI-5P
AUSCA2753-12|Idaea|COI-5P
PHSAU454-12|Idaea|COI-5P
PHLCD851-12|Idaea|COI-5P
WALPC1549-14|Idaea EF20|COI-5P
WALPC1551-14|Idaea EF20|COI-5P
PHSAU667-12|Idaea|COI-5P
PHSAU807-12|Idaea|COI-5P
PHSAU051-12|Idaea|COI-5P
GWORL935-09|Idaea politaria|COI-5P|GU686572
GWOSN330-11|Idaea politaria|COI-5P|KF807105
GWORL760-09|Idaea politaria|COI-5P|HM422578
GWORE1177-08|Idaea politaria|COI-5P
GWORL759-09|Idaea politaria|COI-5P|HM376809
GWORC956-08|Idaea politaria|COI-5P
GWORC957-08|Idaea politaria|COI-5P
GWORC958-08|Idaea politaria|COI-5P
GWORD673-08|Idaea politaria|COI-5P|KF807211
GWORD675-08|Idaea politaria|COI-5P|KF807249
GWOSP902-11|Idaea politaria|COI-5P|KF808129
GWORC1168-08|Idaea politaria|COI-5P|KF807435
GWORD674-08|Idaea politaria|COI-5P|KF807384
GWOTI453-12|Idaea fuscovenosa|COI-5P|KF807518
LENOA813-11|Idaea fuscovenosa|COI-5P|KF808066
GWOSP723-11|Idaea fuscovenosa|COI-5P|KF807799
CGUKC579-09|Idaea fuscovenosa|COI-5P|KF807305
CGUKB536-09|Idaea fuscovenosa|COI-5P|KF807774
CGUKB436-09|Idaea fuscovenosa|COI-5P|KF807789
GBLAF410-14|Idaea fuscovenosa|COI-5P
LENOA814-11|Idaea fuscovenosa|COI-5P|KF807992
CGUKB521-09|Idaea fuscovenosa|COI-5P|KF807737
CGUKB547-09|Idaea fuscovenosa|COI-5P|KF807907
CGUKB554-09|Idaea fuscovenosa|COI-5P|KF807723
ANBIO116-19|Idaea fuscovenosa|COI-5P
GBLAC1091-13|Idaea fuscovenosa|COI-5P
LENOA817-11|Idaea fuscovenosa|COI-5P|KF807455
GWOR4430-09|Idaea fuscovenosaAH01Mr|COI-5P

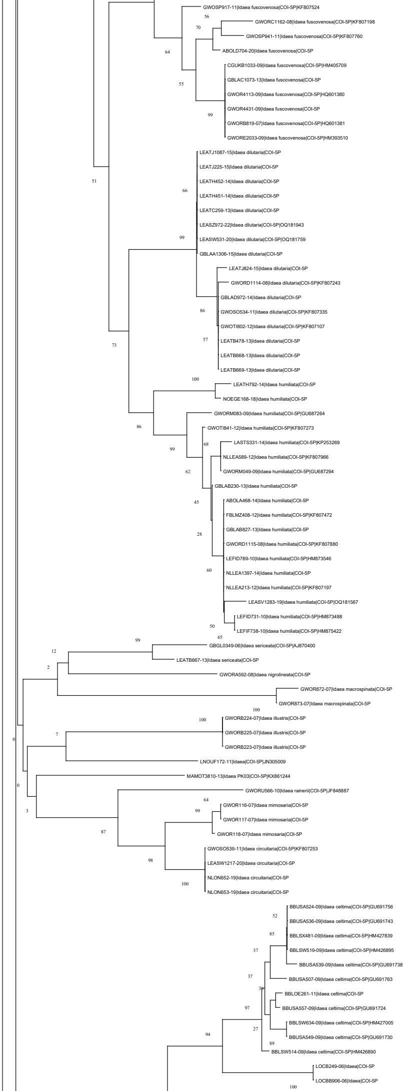
GWOSP917-11|Idaea fuscovenosa|COI-5P|KF807524
GWORC1162-08|Idaea fuscovenosa|COI-5P|KF807198
GWOSP941-11|Idaea fuscovenosa|COI-5P|KF807760
ABOLD704-20|Idaea fuscovenosa|COI-5P
CGUKB1033-09|Idaea fuscovenosa|COI-5P|HM405709
GBLAC1073-13|Idaea fuscovenosa|COI-5P
GWOR4113-09|Idaea fuscovenosa|COI-5P|HQ601380
GWOR4431-09|Idaea fuscovenosa|COI-5P
GWORB819-07|Idaea fuscovenosa|COI-5P|HQ601381
GWORE2033-09|Idaea fuscovenosa|COI-5P|HM393510
LEATJ1087-15|Idaea dilutaria|COI-5P
LEATJ225-15|Idaea dilutaria|COI-5P
LEATH452-14|Idaea dilutaria|COI-5P
LEATH451-14|Idaea dilutaria|COI-5P
LEATC259-13|Idaea dilutaria|COI-5P
LEASZ972-22|Idaea dilutaria|COI-5P|OQ181943
LEASW531-20|Idaea dilutaria|COI-5P|OQ181759
GBLAA1306-15|Idaea dilutaria|COI-5P
LEATJ824-15|Idaea dilutaria|COI-5P
GWORD1114-08|Idaea dilutaria|COI-5P|KF807243
GBLAD972-14|Idaea dilutaria|COI-5P
GWOSO534-11|Idaea dilutaria|COI-5P|KF807335
GWOTI802-12|Idaea dilutaria|COI-5P|KF807107
LEATB478-13|Idaea dilutaria|COI-5P
LEATB668-13|Idaea dilutaria|COI-5P
LEATB669-13|Idaea dilutaria|COI-5P
LEATH792-14|Idaea humiliata|COI-5P
NOEGE168-18|Idaea humiliata|COI-5P
GWORM083-09|Idaea humiliata|COI-5P|GU687264
GWOTI841-12|Idaea humiliata|COI-5P|KF807273
LASTS331-14|Idaea humiliata|COI-5P|KP253269
NLLEA589-12|Idaea humiliata|COI-5P|KF807966
GWORM049-09|Idaea humiliata|COI-5P|GU687294
GBLAB230-13|Idaea humiliata|COI-5P
ABOLA468-14|Idaea humiliata|COI-5P
FBLMZ408-12|Idaea humiliata|COI-5P|KF807472
GBLAB827-13|Idaea humiliata|COI-5P
GWORD1115-08|Idaea humiliata|COI-5P|KF807880
LEFID789-10|Idaea humiliata|COI-5P|HM873546
NLLEA1397-14|Idaea humiliata|COI-5P
NLLEA213-12|Idaea humiliata|COI-5P|KF807197
LEASV1283-19|Idaea humiliata|COI-5P|OQ181567
LEFID731-10|Idaea humiliata|COI-5P|HM873488
LEFIF738-10|Idaea humiliata|COI-5P|HM875422
GBGL0349-06|Idaea sericeata|COI-5P|AJ870400
LEATB667-13|Idaea sericeata|COI-5P
GWORA592-08|Idaea nigrolineata|COI-5P
GWOR872-07|Idaea macrospinata|COI-5P
GWOR873-07|Idaea macrospinata|COI-5P
GWORB224-07|Idaea illustris|COI-5P
GWORB225-07|Idaea illustris|COI-5P
GWORB223-07|Idaea illustris|COI-5P
LNOUF172-11|Idaea|COI-5P|JN305009
MAMOT3810-13|Idaea PK03|COI-5P|KX861244
GWORU566-10|Idaea rainerii|COI-5P|JF848887
GWOR116-07|Idaea mimosaria|COI-5P
GWOR117-07|Idaea mimosaria|COI-5P
GWOR118-07|Idaea mimosaria|COI-5P
GWOSO539-11|Idaea circuitaria|COI-5P|KF807253
LEASW1217-20|Idaea circuitaria|COI-5P
NLON652-19|Idaea circuitaria|COI-5P
NLON653-19|Idaea circuitaria|COI-5P
BBUSA524-09|Idaea celtima|COI-5P|GU691756
BBUSA536-09|Idaea celtima|COI-5P|GU691743
BBLSX481-09|Idaea celtima|COI-5P|HM427839
BBLSW519-09|Idaea celtima|COI-5P|HM426895
BBUSA539-09|Idaea celtima|COI-5P|GU691738
BBUSA507-09|Idaea celtima|COI-5P|GU691763
BBLOE261-11|Idaea celtima|COI-5P
BBUSA557-09|Idaea celtima|COI-5P|GU691724
BBLSW634-09|Idaea celtima|COI-5P|HM427005
BBUSA549-09|Idaea celtima|COI-5P|GU691730
BBLSW514-09|Idaea celtima|COI-5P|HM426890
LOCB249-06|Idaea|COI-5P
LOCBB906-06|Idaea|COI-5P

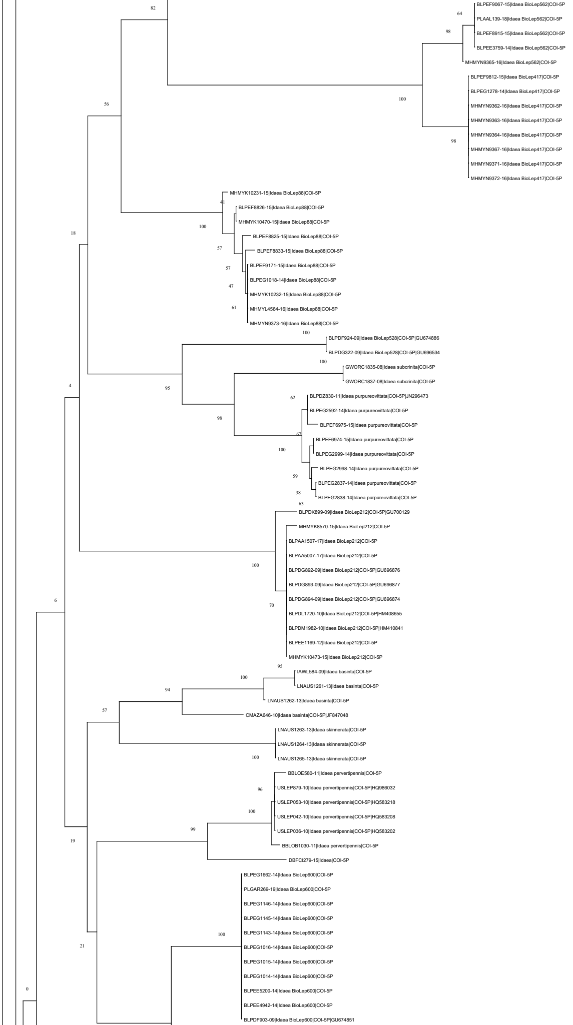
BLPEF9067-15|Idaea BioLep562|COI-5P
PLAAL139-18|Idaea BioLep562|COI-5P
BLPEF8915-15|Idaea BioLep562|COI-5P
BLPEE3759-14|Idaea BioLep562|COI-5P
MHMYN9365-16|Idaea BioLep562|COI-5P
BLPEF9812-15|Idaea BioLep417|COI-5P
BLPEG1278-14|Idaea BioLep417|COI-5P
MHMYN9362-16|Idaea BioLep417|COI-5P
MHMYN9363-16|Idaea BioLep417|COI-5P
MHMYN9364-16|Idaea BioLep417|COI-5P
MHMYN9367-16|Idaea BioLep417|COI-5P
MHMYN9371-16|Idaea BioLep417|COI-5P
MHMYN9372-16|Idaea BioLep417|COI-5P
MHMYK10231-15|Idaea BioLep88|COI-5P
BLPEF8826-15|Idaea BioLep88|COI-5P
MHMYK10470-15|Idaea BioLep88|COI-5P
BLPEF8825-15|Idaea BioLep88|COI-5P
BLPEF8833-15|Idaea BioLep88|COI-5P
BLPEF9171-15|Idaea BioLep88|COI-5P
BLPEG1018-14|Idaea BioLep88|COI-5P
MHMYK10232-15|Idaea BioLep88|COI-5P
MHMYL4584-16|Idaea BioLep88|COI-5P
MHMYN9373-16|Idaea BioLep88|COI-5P
BLPDF924-09|Idaea BioLep528|COI-5P|GU674886
BLPDG322-09|Idaea BioLep528|COI-5P|GU696534
GWORC1835-08|Idaea subcrinita|COI-5P
GWORC1837-08|Idaea subcrinita|COI-5P
BLPDZ830-11|Idaea purpureovittata|COI-5P|JN296473
BLPEG2592-14|Idaea purpureovittata|COI-5P
BLPEF6975-15|Idaea purpureovittata|COI-5P
BLPEF6974-15|Idaea purpureovittata|COI-5P
BLPEG2999-14|Idaea purpureovittata|COI-5P
BLPEG2998-14|Idaea purpureovittata|COI-5P
BLPEG2837-14|Idaea purpureovittata|COI-5P
BLPEG2838-14|Idaea purpureovittata|COI-5P
BLPDK899-09|Idaea BioLep212|COI-5P|GU700129
MHMYK8570-15|Idaea BioLep212|COI-5P
BLPAA1507-17|Idaea BioLep212|COI-5P
BLPAA5007-17|Idaea BioLep212|COI-5P
BLPDG892-09|Idaea BioLep212|COI-5P|GU696876
BLPDG893-09|Idaea BioLep212|COI-5P|GU696877
BLPDG894-09|Idaea BioLep212|COI-5P|GU696874
BLPDL1720-10|Idaea BioLep212|COI-5P|HM408655
BLPDM1982-10|Idaea BioLep212|COI-5P|HM410841
BLPEE1169-12|Idaea BioLep212|COI-5P
MHMYK10473-15|Idaea BioLep212|COI-5P
IAWL584-09|Idaea basinta|COI-5P
LNAUS1261-13|Idaea basinta|COI-5P
LNAUS1262-13|Idaea basinta|COI-5P
CMAZA646-10|Idaea basinta|COI-5P|JF847048
LNAUS1263-13|Idaea skinnerata|COI-5P
LNAUS1264-13|Idaea skinnerata|COI-5P
LNAUS1265-13|Idaea skinnerata|COI-5P
BBLOE580-11|Idaea pervertipennis|COI-5P
USLEP879-10|Idaea pervertipennis|COI-5P|HQ986032
USLEP053-10|Idaea pervertipennis|COI-5P|HQ583218
USLEP042-10|Idaea pervertipennis|COI-5P|HQ583208
USLEP036-10|Idaea pervertipennis|COI-5P|HQ583202
BBLOB1030-11|Idaea pervertipennis|COI-5P
DBFCI279-15|Idaea|COI-5P
BLPEG1662-14|Idaea BioLep600|COI-5P
PLGAR269-19|Idaea BioLep600|COI-5P
BLPEG1146-14|Idaea BioLep600|COI-5P
BLPEG1145-14|Idaea BioLep600|COI-5P
BLPEG1143-14|Idaea BioLep600|COI-5P
BLPEG1016-14|Idaea BioLep600|COI-5P
BLPEG1015-14|Idaea BioLep600|COI-5P
BLPEG1014-14|Idaea BioLep600|COI-5P
BLPEE5200-14|Idaea BioLep600|COI-5P
BLPEE4942-14|Idaea BioLep600|COI-5P
BLPDF903-09|Idaea BioLep600|COI-5P|GU674851

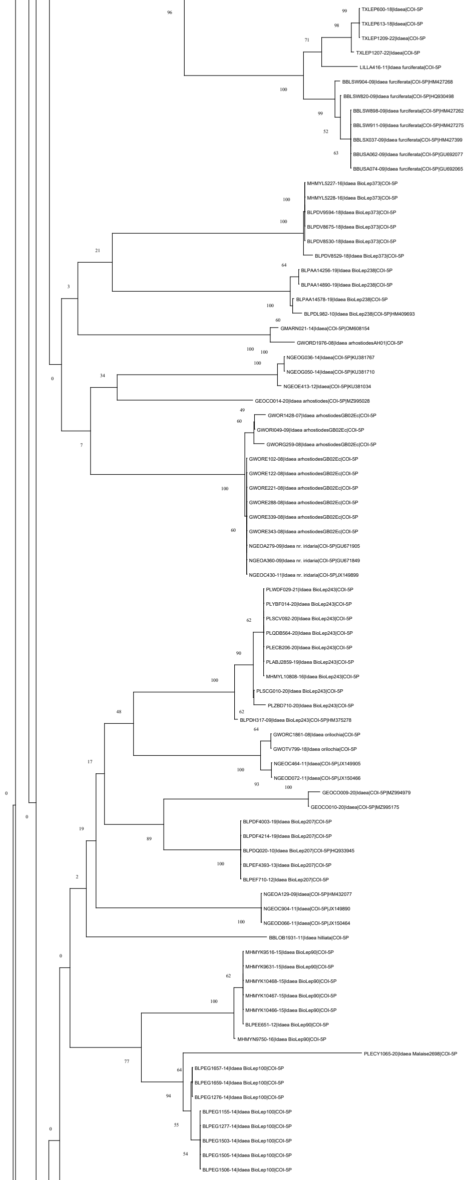
TXLEP600-18|Idaea|COI-5P
TXLEP613-18|Idaea|COI-5P
TXLEP1209-22|Idaea|COI-5P
TXLEP1207-22|Idaea|COI-5P
LILLA416-11|Idaea furciferata|COI-5P
BBLSW904-09|Idaea furciferata|COI-5P|HM427268
BBLSW820-09|Idaea furciferata|COI-5P|HQ930498
BBLSW898-09|Idaea furciferata|COI-5P|HM427262
BBLSW911-09|Idaea furciferata|COI-5P|HM427275
BBLSX037-09|Idaea furciferata|COI-5P|HM427399
BBUSA062-09|Idaea furciferata|COI-5P|GU692077
BBUSA074-09|Idaea furciferata|COI-5P|GU692065
MHMYL5227-16|Idaea BioLep373|COI-5P
MHMYL5228-16|Idaea BioLep373|COI-5P
BLPDV9594-18|Idaea BioLep373|COI-5P
BLPDV8675-18|Idaea BioLep373|COI-5P
BLPDV8530-18|Idaea BioLep373|COI-5P
BLPDV8529-18|Idaea BioLep373|COI-5P
BLPAA14256-19|Idaea BioLep238|COI-5P
BLPAA14890-19|Idaea BioLep238|COI-5P
BLPAA14578-19|Idaea BioLep238|COI-5P
BLPDL982-10|Idaea BioLep238|COI-5P|HM409693
GMARN021-14|Idaea|COI-5P|OM608154
GWORD1976-08|Idaea arhostiodesAH01|COI-5P
NGEOG036-14|Idaea|COI-5P|KU381767
NGEOG050-14|Idaea|COI-5P|KU381710
NGEOE413-12|Idaea|COI-5P|KU381034
GEOCO014-20|Idaea arhostiodes|COI-5P|MZ995028
GWOR1428-07|Idaea arhostiodesGB02Ec|COI-5P
GWORI049-09|Idaea arhostiodesGB02Ec|COI-5P
GWORG259-08|Idaea arhostiodesGB02Ec|COI-5P
GWORE102-08|Idaea arhostiodesGB02Ec|COI-5P
GWORE122-08|Idaea arhostiodesGB02Ec|COI-5P
GWORE221-08|Idaea arhostiodesGB02Ec|COI-5P
GWORE288-08|Idaea arhostiodesGB02Ec|COI-5P
GWORE339-08|Idaea arhostiodesGB02Ec|COI-5P
GWORE343-08|Idaea arhostiodesGB02Ec|COI-5P
NGEOA279-09|Idaea nr. iridaria|COI-5P|GU671905
NGEOA360-09|Idaea nr. iridaria|COI-5P|GU671849
NGEOC430-11|Idaea nr. iridaria|COI-5P|JX149899
PLWDF029-21|Idaea BioLep243|COI-5P
PLYBF014-20|Idaea BioLep243|COI-5P
PLSCV092-20|Idaea BioLep243|COI-5P
PLQDB564-20|Idaea BioLep243|COI-5P
PLECB206-20|Idaea BioLep243|COI-5P
PLABJ2859-19|Idaea BioLep243|COI-5P
MHMYL10808-16|Idaea BioLep243|COI-5P
PLSCG010-20|Idaea BioLep243|COI-5P
PLZBD710-20|Idaea BioLep243|COI-5P
BLPDH317-09|Idaea BioLep243|COI-5P|HM375278
GWORC1861-08|Idaea orilochia|COI-5P
GWOTV799-18|Idaea orilochia|COI-5P
NGEOC464-11|Idaea|COI-5P|JX149905
NGEOD072-11|Idaea|COI-5P|JX150466
GEOCO009-20|Idaea|COI-5P|MZ994979
GEOCO010-20|Idaea|COI-5P|MZ995175
BLPDF4003-19|Idaea BioLep207|COI-5P
BLPDF4214-19|Idaea BioLep207|COI-5P
BLPDQ020-10|Idaea BioLep207|COI-5P|HQ933945
BLPEF4393-13|Idaea BioLep207|COI-5P
BLPEF710-12|Idaea BioLep207|COI-5P
NGEOA129-09|Idaea|COI-5P|HM432077
NGEOC904-11|Idaea|COI-5P|JX149890
NGEOD066-11|Idaea|COI-5P|JX150464
BBLOB1931-11|Idaea hilliata|COI-5P
MHMYK9516-15|Idaea BioLep90|COI-5P
MHMYK9631-15|Idaea BioLep90|COI-5P
MHMYK10468-15|Idaea BioLep90|COI-5P
MHMYK10467-15|Idaea BioLep90|COI-5P
MHMYK10466-15|Idaea BioLep90|COI-5P
BLPEE651-12|Idaea BioLep90|COI-5P
MHMYN9750-16|Idaea BioLep90|COI-5P
PLECY1065-20|Idaea Malaise2698|COI-5P
BLPEG1657-14|Idaea BioLep100|COI-5P
BLPEG1659-14|Idaea BioLep100|COI-5P
BLPEG1276-14|Idaea BioLep100|COI-5P
BLPEG1155-14|Idaea BioLep100|COI-5P
BLPEG1277-14|Idaea BioLep100|COI-5P
BLPEG1503-14|Idaea BioLep100|COI-5P
BLPEG1505-14|Idaea BioLep100|COI-5P
BLPEG1506-14|Idaea BioLep100|COI-5P

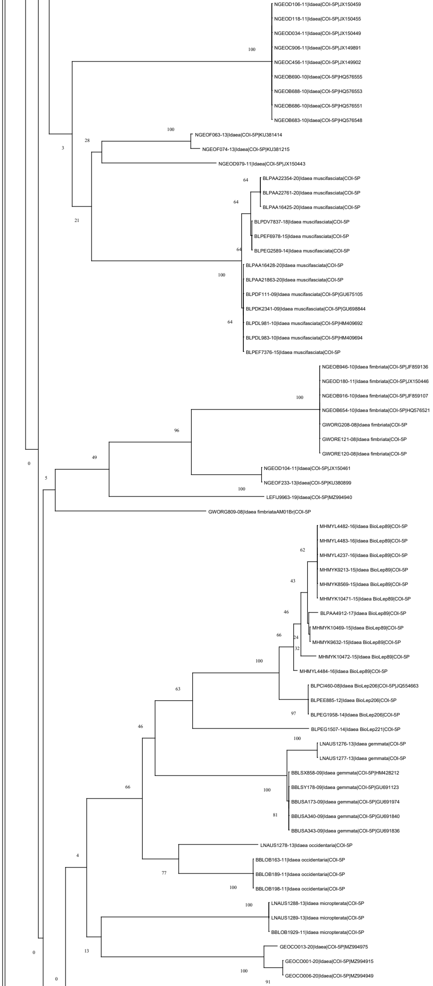
NGEOD106-11|Idaea|COI-5P|JX150459
NGEOD118-11|Idaea|COI-5P|JX150455
NGEOD034-11|Idaea|COI-5P|JX150449
100
NGEOC906-11|Idaea|COI-5P|JX149891
NGEOC456-11|Idaea|COI-5P|JX149902
NGEOB690-10|Idaea|COI-5P|HQ576555
NGEOB688-10|Idaea|COI-5P|HQ576553
NGEOB686-10|Idaea|COI-5P|HQ576551
NGEOB683-10|Idaea|COI-5P|HQ576548
100
NGEOF063-13|Idaea|COI-5P|KU381414
28
3
NGEOF074-13|Idaea|COI-5P|KU381215
NGEOD979-11|Idaea|COI-5P|JX150443
64
BLPAA22354-20|Idaea muscifasciata|COI-5P
BLPAA22761-20|Idaea muscifasciata|COI-5P
64
BLPAA16425-20|Idaea muscifasciata|COI-5P
21
BLPDV7837-18|Idaea muscifasciata|COI-5P
BLPEF6978-15|Idaea muscifasciata|COI-5P
64
BLPEG2589-14|Idaea muscifasciata|COI-5P
BLPAA16428-20|Idaea muscifasciata|COI-5P
100
BLPAA21863-20|Idaea muscifasciata|COI-5P
BLPDF111-09|Idaea muscifasciata|COI-5P|GU675105
BLPDK2341-09|Idaea muscifasciata|COI-5P|GU698844
64
BLPDL981-10|Idaea muscifasciata|COI-5P|HM409692
BLPDL983-10|Idaea muscifasciata|COI-5P|HM409694
BLPEF7376-15|Idaea muscifasciata|COI-5P
NGEOB946-10|Idaea fimbriata|COI-5P|JF859136
NGEOD180-11|Idaea fimbriata|COI-5P|JX150446
100
NGEOB916-10|Idaea fimbriata|COI-5P|JF859107
NGEOB654-10|Idaea fimbriata|COI-5P|HQ576521
GWORG208-08|Idaea fimbriata|COI-5P
96
GWORE121-08|Idaea fimbriata|COI-5P
GWORE120-08|Idaea fimbriata|COI-5P
49
0
NGEOD104-11|Idaea|COI-5P|JX150461
5
NGEOF233-13|Idaea|COI-5P|KU380899
100
LEFIJ9963-19|Idaea|COI-5P|MZ994940
GWORG809-08|Idaea fimbriataAM01Br|COI-5P
MHMYL4482-16|Idaea BioLep89|COI-5P
MHMYL4483-16|Idaea BioLep89|COI-5P
62
MHMYL4237-16|Idaea BioLep89|COI-5P
MHMYK9213-15|Idaea BioLep89|COI-5P
43
MHMYK8569-15|Idaea BioLep89|COI-5P
MHMYK10471-15|Idaea BioLep89|COI-5P
46
BLPAA4912-17|Idaea BioLep89|COI-5P
MHMYK10469-15|Idaea BioLep89|COI-5P
66
24
MHMYK9632-15|Idaea BioLep89|COI-5P
32
MHMYK10472-15|Idaea BioLep89|COI-5P
100
MHMYL4484-16|Idaea BioLep89|COI-5P
63
BLPCI460-08|Idaea BioLep206|COI-5P|JQ554663
BLPEE885-12|Idaea BioLep206|COI-5P
97
BLPEG1958-14|Idaea BioLep206|COI-5P
46
BLPEG1507-14|Idaea BioLep221|COI-5P
100
LNAUS1276-13|Idaea gemmata|COI-5P
LNAUS1277-13|Idaea gemmata|COI-5P
BBLSX858-09|Idaea gemmata|COI-5P|HM428212
66
100
BBLSY178-09|Idaea gemmata|COI-5P|GU691123
BBUSA173-09|Idaea gemmata|COI-5P|GU691974
81
BBUSA340-09|Idaea gemmata|COI-5P|GU691840
BBUSA343-09|Idaea gemmata|COI-5P|GU691836
LNAUS1278-13|Idaea occidentaria|COI-5P
4
BBLOB163-11|Idaea occidentaria|COI-5P
77
BBLOB189-11|Idaea occidentaria|COI-5P
100
BBLOB198-11|Idaea occidentaria|COI-5P
100
LNAUS1288-13|Idaea micropterata|COI-5P
LNAUS1289-13|Idaea micropterata|COI-5P
BBLOB1929-11|Idaea micropterata|COI-5P
GEOCO013-20|Idaea|COI-5P|MZ994975
0
13
GEOCO001-20|Idaea|COI-5P|MZ994915
100
GEOCO006-20|Idaea|COI-5P|MZ994949
0
91

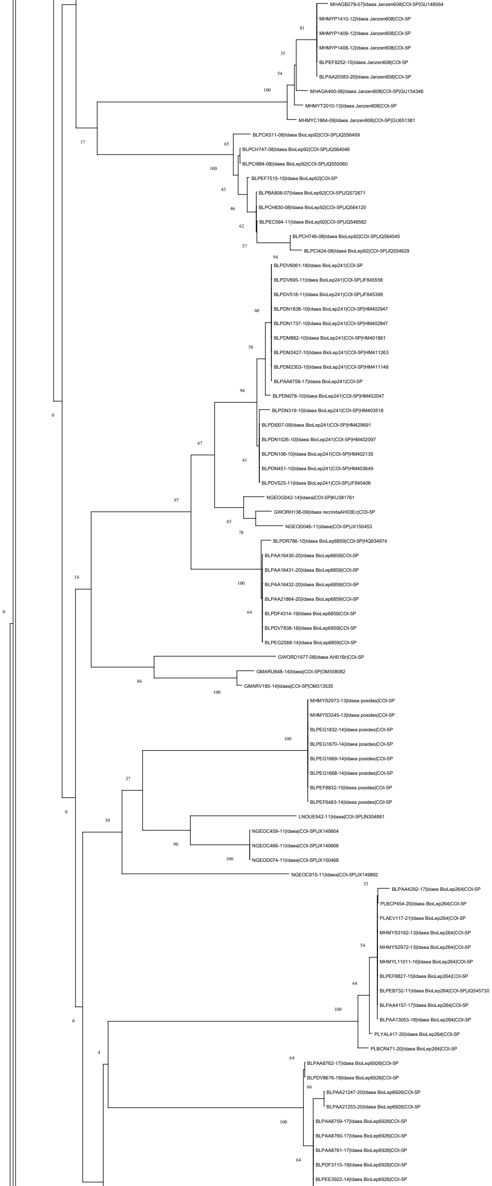
MHAGB278-07|Idaea Janzen608|COI-5P|GU148564
MHMYP1410-12|Idaea Janzen608|COI-5P
MHMYP1409-12|Idaea Janzen608|COI-5P
MHMYP1408-12|Idaea Janzen608|COI-5P
BLPEF8252-15|Idaea Janzen608|COI-5P
BLPAA20583-20|Idaea Janzen608|COI-5P
MHAGA400-06|Idaea Janzen608|COI-5P|GU154346
MHMYT2010-13|Idaea Janzen608|COI-5P
MHMYC1864-09|Idaea Janzen608|COI-5P|GU651381
BLPCK511-08|Idaea BioLep92|COI-5P|JQ556499
BLPCH747-08|Idaea BioLep92|COI-5P|JQ564046
BLPCI884-08|Idaea BioLep92|COI-5P|JQ555060
BLPEF7515-15|Idaea BioLep92|COI-5P
BLPBA808-07|Idaea BioLep92|COI-5P|JQ572671
BLPCH830-08|Idaea BioLep92|COI-5P|JQ564120
BLPEC594-11|Idaea BioLep92|COI-5P|JQ546582
BLPCH746-08|Idaea BioLep92|COI-5P|JQ564045
BLPCI424-08|Idaea BioLep92|COI-5P|JQ554629
BLPDV6061-18|Idaea BioLep241|COI-5P
BLPDV695-11|Idaea BioLep241|COI-5P|JF845556
BLPDV518-11|Idaea BioLep241|COI-5P|JF845399
BLPDN1838-10|Idaea BioLep241|COI-5P|HM402947
BLPDN1737-10|Idaea BioLep241|COI-5P|HM402847
BLPDM882-10|Idaea BioLep241|COI-5P|HM401861
BLPDM2427-10|Idaea BioLep241|COI-5P|HM411263
BLPDM2303-10|Idaea BioLep241|COI-5P|HM411146
BLPAA8758-17|Idaea BioLep241|COI-5P
BLPDN078-10|Idaea BioLep241|COI-5P|HM402047
BLPDN319-10|Idaea BioLep241|COI-5P|HM403518
BLPDI007-09|Idaea BioLep241|COI-5P|HM429691
BLPDN1026-10|Idaea BioLep241|COI-5P|HM402097
BLPDN106-10|Idaea BioLep241|COI-5P|HM402135
BLPDN451-10|Idaea BioLep241|COI-5P|HM403649
BLPDV525-11|Idaea BioLep241|COI-5P|JF845406
NGEOG042-14|Idaea|COI-5P|KU381761
GWORH138-09|Idaea recrinitaAH03Ec|COI-5P
NGEOD046-11|Idaea|COI-5P|JX150453
BLPDR786-10|Idaea BioLep6859|COI-5P|HQ934974
BLPAA16430-20|Idaea BioLep6859|COI-5P
BLPAA16431-20|Idaea BioLep6859|COI-5P
BLPAA16432-20|Idaea BioLep6859|COI-5P
BLPAA21864-20|Idaea BioLep6859|COI-5P
BLPDF4314-19|Idaea BioLep6859|COI-5P
BLPDV7838-18|Idaea BioLep6859|COI-5P
BLPEG2588-14|Idaea BioLep6859|COI-5P
GWORD1977-08|Idaea AH01Br|COI-5P
GMARU648-14|Idaea|COI-5P|OM558082
GMARV185-14|Idaea|COI-5P|OM313535
MHMYS2973-13|Idaea posides|COI-5P
MHMYS3345-13|Idaea posides|COI-5P
BLPEG1832-14|Idaea posides|COI-5P
BLPEG1670-14|Idaea posides|COI-5P
BLPEG1669-14|Idaea posides|COI-5P
BLPEG1668-14|Idaea posides|COI-5P
BLPEF8832-15|Idaea posides|COI-5P
BLPEF6483-14|Idaea posides|COI-5P
LNOUE942-11|Idaea|COI-5P|JN304881
NGEOC459-11|Idaea|COI-5P|JX149904
NGEOC466-11|Idaea|COI-5P|JX149906
NGEOD074-11|Idaea|COI-5P|JX150468
NGEOC915-11|Idaea|COI-5P|JX149892
BLPAA4292-17|Idaea BioLep264|COI-5P
PLBCP454-20|Idaea BioLep264|COI-5P
PLAEV117-21|Idaea BioLep264|COI-5P
MHMYS3162-13|Idaea BioLep264|COI-5P
MHMYS2972-13|Idaea BioLep264|COI-5P
MHMYL11011-16|Idaea BioLep264|COI-5P
BLPEF8827-15|Idaea BioLep264|COI-5P
BLPEB732-11|Idaea BioLep264|COI-5P|JQ545730
BLPAA4157-17|Idaea BioLep264|COI-5P
BLPAA13053-18|Idaea BioLep264|COI-5P
PLYAL417-20|Idaea BioLep264|COI-5P
PLBCR471-20|Idaea BioLep264|COI-5P
BLPAA8762-17|Idaea BioLep6926|COI-5P
BLPDV8676-18|Idaea BioLep6926|COI-5P
BLPAA21247-20|Idaea BioLep6926|COI-5P
BLPAA21253-20|Idaea BioLep6926|COI-5P
BLPAA8759-17|Idaea BioLep6926|COI-5P
BLPAA8760-17|Idaea BioLep6926|COI-5P
BLPAA8761-17|Idaea BioLep6926|COI-5P
BLPDF3115-19|Idaea BioLep6926|COI-5P
BLPEE3922-14|Idaea BioLep6926|COI-5P

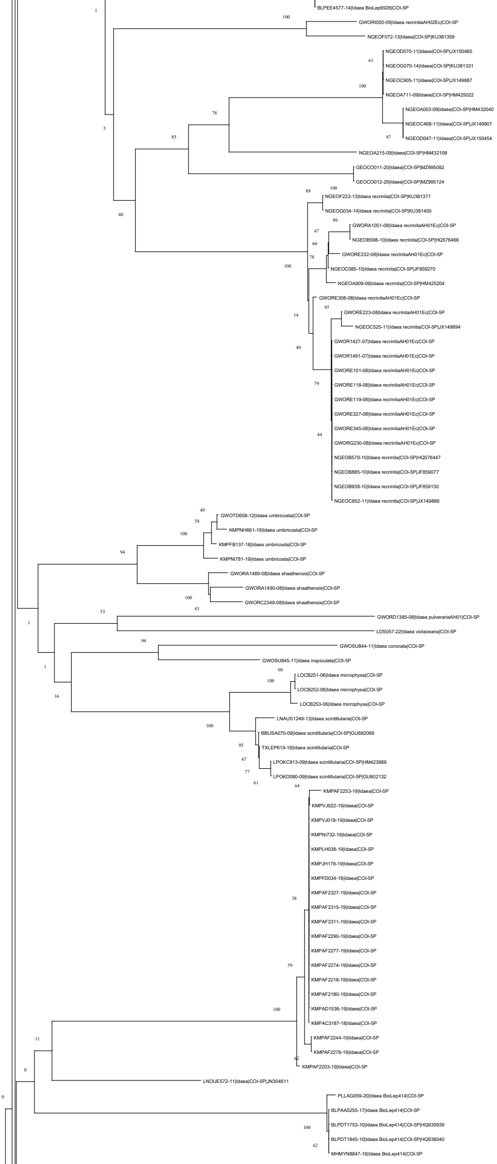
BLPEE4577-14|Idaea BioLep6926|COI-5P
GWORI050-09|Idaea recrinitaAH02Ec|COI-5P
NGEOF072-13|Idaea|COI-5P|KU381359
NGEOD070-11|Idaea|COI-5P|JX150465
NGEOG070-14|Idaea|COI-5P|KU381331
NGEOC905-11|Idaea|COI-5P|JX149887
NGEOA711-09|Idaea|COI-5P|HM425022
NGEOA003-09|Idaea|COI-5P|HM432040
NGEOC468-11|Idaea|COI-5P|JX149907
NGEOD047-11|Idaea|COI-5P|JX150454
NGEOA215-09|Idaea|COI-5P|HM432108
GEOCO011-20|Idaea|COI-5P|MZ995092
GEOCO012-20|Idaea|COI-5P|MZ995124
NGEOF222-13|Idaea recrinita|COI-5P|KU381371
NGEOG034-14|Idaea recrinita|COI-5P|KU381450
GWORA1051-08|Idaea recrinitaAH01Ec|COI-5P
NGEOB598-10|Idaea recrinita|COI-5P|HQ576466
GWORE222-08|Idaea recrinitaAH01Ec|COI-5P
NGEOC085-10|Idaea recrinita|COI-5P|JF859270
NGEOA909-09|Idaea recrinita|COI-5P|HM425204
GWORE308-08|Idaea recrinitaAH01Ec|COI-5P
GWORE223-08|Idaea recrinitaAH01Ec|COI-5P
NGEOC525-11|Idaea recrinita|COI-5P|JX149894
GWOR1427-07|Idaea recrinitaAH01Ec|COI-5P
GWOR1491-07|Idaea recrinitaAH01Ec|COI-5P
GWORE101-08|Idaea recrinitaAH01Ec|COI-5P
GWORE118-08|Idaea recrinitaAH01Ec|COI-5P
GWORE119-08|Idaea recrinitaAH01Ec|COI-5P
GWORE327-08|Idaea recrinitaAH01Ec|COI-5P
GWORE345-08|Idaea recrinitaAH01Ec|COI-5P
GWORG230-08|Idaea recrinitaAH01Ec|COI-5P
NGEOB579-10|Idaea recrinita|COI-5P|HQ576447
NGEOB885-10|Idaea recrinita|COI-5P|JF859077
NGEOB939-10|Idaea recrinita|COI-5P|JF859130
NGEOC852-11|Idaea recrinita|COI-5P|JX149889
GWOTD658-12|Idaea umbricosta|COI-5P
KMPNH661-19|Idaea umbricosta|COI-5P
KMPFB137-18|Idaea umbricosta|COI-5P
KMPNI781-19|Idaea umbricosta|COI-5P
GWORA1489-08|Idaea shaathensis|COI-5P
GWORA1490-08|Idaea shaathensis|COI-5P
GWORC2349-08|Idaea shaathensis|COI-5P
GWORD1385-08|Idaea pulverariaAH01|COI-5P
LDS057-22|Idaea violacearia|COI-5P
GWOSU844-11|Idaea coronata|COI-5P
GWOSU845-11|Idaea inspiculata|COI-5P
LOCB251-06|Idaea microphysa|COI-5P
LOCB252-06|Idaea microphysa|COI-5P
LOCB253-06|Idaea microphysa|COI-5P
LNAUS1249-13|Idaea scintillularia|COI-5P
BBUSA070-09|Idaea scintillularia|COI-5P|GU692069
TXLEP619-18|Idaea scintillularia|COI-5P
LPOKC913-09|Idaea scintillularia|COI-5P|HM423986
LPOKD080-09|Idaea scintillularia|COI-5P|GU802132
KMPAF2253-19|Idaea|COI-5P
KMPVJ022-19|Idaea|COI-5P
KMPVJ018-19|Idaea|COI-5P
KMPNI732-19|Idaea|COI-5P
KMPLH038-19|Idaea|COI-5P
KMPJH179-19|Idaea|COI-5P
KMPFD034-18|Idaea|COI-5P
KMPAF2327-19|Idaea|COI-5P
KMPAF2315-19|Idaea|COI-5P
KMPAF2311-19|Idaea|COI-5P
KMPAF2290-19|Idaea|COI-5P
KMPAF2277-19|Idaea|COI-5P
KMPAF2274-19|Idaea|COI-5P
KMPAF2218-19|Idaea|COI-5P
KMPAF2180-19|Idaea|COI-5P
KMPAD1536-19|Idaea|COI-5P
KMPAC3187-18|Idaea|COI-5P
KMPAF2244-19|Idaea|COI-5P
KMPAF2276-19|Idaea|COI-5P
KMPAF2203-19|Idaea|COI-5P
LNOUE572-11|Idaea|COI-5P|JN304611
PLLAG059-20|Idaea BioLep414|COI-5P
BLPAA5255-17|Idaea BioLep414|COI-5P
BLPDT1753-10|Idaea BioLep414|COI-5P|HQ935939
BLPDT1845-10|Idaea BioLep414|COI-5P|HQ936040
MHMYN8847-16|Idaea BioLep414|COI-5P

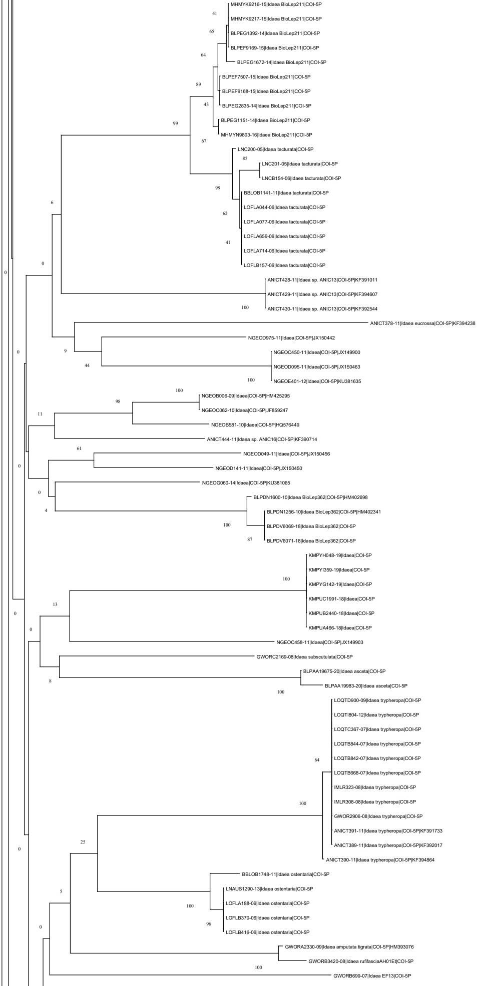
MHMYK9216-15|Idaea BioLep211|COI-5P
MHMYK9217-15|Idaea BioLep211|COI-5P
BLPEG1392-14|Idaea BioLep211|COI-5P
BLPEF9169-15|Idaea BioLep211|COI-5P
BLPEG1672-14|Idaea BioLep211|COI-5P
BLPEF7507-15|Idaea BioLep211|COI-5P
BLPEF9168-15|Idaea BioLep211|COI-5P
BLPEG2835-14|Idaea BioLep211|COI-5P
BLPEG1151-14|Idaea BioLep211|COI-5P
MHMYN9803-16|Idaea BioLep211|COI-5P
LNC200-05|Idaea tacturata|COI-5P
LNC201-05|Idaea tacturata|COI-5P
LNCB154-06|Idaea tacturata|COI-5P
BBLOB1141-11|Idaea tacturata|COI-5P
LOFLA044-06|Idaea tacturata|COI-5P
LOFLA077-06|Idaea tacturata|COI-5P
LOFLA659-06|Idaea tacturata|COI-5P
LOFLA714-06|Idaea tacturata|COI-5P
LOFLB157-06|Idaea tacturata|COI-5P
ANICT428-11|Idaea sp. ANIC13|COI-5P|KF391011
ANICT429-11|Idaea sp. ANIC13|COI-5P|KF394607
ANICT430-11|Idaea sp. ANIC13|COI-5P|KF392544
ANICT378-11|Idaea eucrossa|COI-5P|KF394238
NGEOD975-11|Idaea|COI-5P|JX150442
NGEOC450-11|Idaea|COI-5P|JX149900
NGEOD095-11|Idaea|COI-5P|JX150463
NGEOE401-12|Idaea|COI-5P|KU381635
NGEOB006-09|Idaea|COI-5P|HM425295
NGEOC062-10|Idaea|COI-5P|JF859247
NGEOB581-10|Idaea|COI-5P|HQ576449
ANICT444-11|Idaea sp. ANIC16|COI-5P|KF390714
NGEOD049-11|Idaea|COI-5P|JX150456
NGEOD141-11|Idaea|COI-5P|JX150450
NGEOG060-14|Idaea|COI-5P|KU381065
BLPDN1600-10|Idaea BioLep362|COI-5P|HM402698
BLPDN1256-10|Idaea BioLep362|COI-5P|HM402341
BLPDV6069-18|Idaea BioLep362|COI-5P
BLPDV6071-18|Idaea BioLep362|COI-5P
KMPYH048-19|Idaea|COI-5P
KMPYI359-19|Idaea|COI-5P
KMPYG142-19|Idaea|COI-5P
KMPUC1991-18|Idaea|COI-5P
KMPUB2440-18|Idaea|COI-5P
KMPUA466-18|Idaea|COI-5P
NGEOC458-11|Idaea|COI-5P|JX149903
GWORC2169-08|Idaea subscutulata|COI-5P
BLPAA19675-20|Idaea asceta|COI-5P
BLPAA19983-20|Idaea asceta|COI-5P
LOQTD900-09|Idaea trypheropa|COI-5P
LOQTI804-12|Idaea trypheropa|COI-5P
LOQTC367-07|Idaea trypheropa|COI-5P
LOQTB844-07|Idaea trypheropa|COI-5P
LOQTB842-07|Idaea trypheropa|COI-5P
LOQTB668-07|Idaea trypheropa|COI-5P
IMLR323-08|Idaea trypheropa|COI-5P
IMLR308-08|Idaea trypheropa|COI-5P
GWOR2906-08|Idaea trypheropa|COI-5P
ANICT391-11|Idaea trypheropa|COI-5P|KF391733
ANICT389-11|Idaea trypheropa|COI-5P|KF392017
ANICT390-11|Idaea trypheropa|COI-5P|KF394864
BBLOB1748-11|Idaea ostentaria|COI-5P
LNAUS1290-13|Idaea ostentaria|COI-5P
LOFLA188-06|Idaea ostentaria|COI-5P
LOFLB370-06|Idaea ostentaria|COI-5P
LOFLB416-06|Idaea ostentaria|COI-5P
GWORA2330-09|Idaea amputata tigrata|COI-5P|HM393076
GWORB3420-08|Idaea rufifasciaAH01Et|COI-5P
GWORB699-07|Idaea EF13|COI-5P

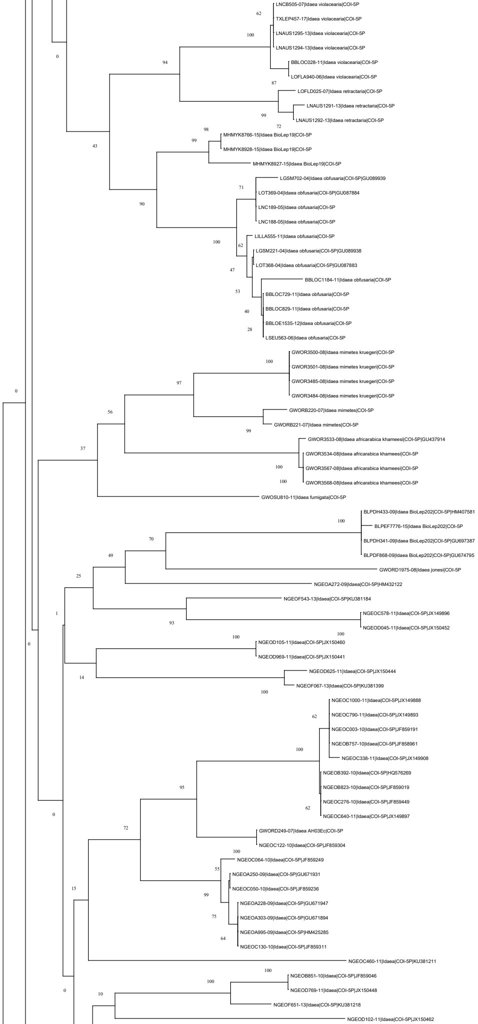
LNCB505-07|Idaea violacearia|COI-5P
62
TXLEP457-17|Idaea violacearia|COI-5P
100
LNAUS1295-13|Idaea violacearia|COI-5P
LNAUS1294-13|Idaea violacearia|COI-5P
0
94
BBLOC028-11|Idaea violacearia|COI-5P
LOFLA940-06|Idaea violacearia|COI-5P
87
LOFLD025-07|Idaea retractaria|COI-5P
LNAUS1291-13|Idaea retractaria|COI-5P
99
LNAUS1292-13|Idaea retractaria|COI-5P
72
98
MHMYK8766-15|Idaea BioLep19|COI-5P
99
43
MHMYK8928-15|Idaea BioLep19|COI-5P
MHMYK8927-15|Idaea BioLep19|COI-5P
LGSM702-04|Idaea obfusaria|COI-5P|GU089939
71
LOT369-04|Idaea obfusaria|COI-5P|GU087884
90
LNC189-05|Idaea obfusaria|COI-5P
LNC188-05|Idaea obfusaria|COI-5P
LILLA555-11|Idaea obfusaria|COI-5P
100
62
LGSM221-04|Idaea obfusaria|COI-5P|GU089938
LOT368-04|Idaea obfusaria|COI-5P|GU087883
47
BBLOC1184-11|Idaea obfusaria|COI-5P
53
BBLOC729-11|Idaea obfusaria|COI-5P
BBLOC829-11|Idaea obfusaria|COI-5P
40
BBLOE1535-12|Idaea obfusaria|COI-5P
28
LSEU563-06|Idaea obfusaria|COI-5P
GWOR3500-08|Idaea mimetes kruegeri|COI-5P
100
GWOR3501-08|Idaea mimetes kruegeri|COI-5P
GWOR3485-08|Idaea mimetes kruegeri|COI-5P
97
0
GWOR3484-08|Idaea mimetes kruegeri|COI-5P
GWORB220-07|Idaea mimetes|COI-5P
56
GWORB221-07|Idaea mimetes|COI-5P
99
GWOR3533-08|Idaea africarabica khameesi|COI-5P|GU437914
37
GWOR3534-08|Idaea africarabica khameesi|COI-5P
100
GWOR3567-08|Idaea africarabica khameesi|COI-5P
100
GWOR3568-08|Idaea africarabica khameesi|COI-5P
GWOSU810-11|Idaea fumigata|COI-5P
BLPDH433-09|Idaea BioLep202|COI-5P|HM407581
100
BLPEF7776-15|Idaea BioLep202|COI-5P
70
BLPDH341-09|Idaea BioLep202|COI-5P|GU697387
49
BLPDF868-09|Idaea BioLep202|COI-5P|GU674795
GWORD1975-08|Idaea jonesi|COI-5P
25
NGEOA272-09|Idaea|COI-5P|HM432122
NGEOF543-13|Idaea|COI-5P|KU381184
1
NGEOC578-11|Idaea|COI-5P|JX149896
93
NGEOD045-11|Idaea|COI-5P|JX150452
100
100
NGEOD105-11|Idaea|COI-5P|JX150460
0
NGEOD969-11|Idaea|COI-5P|JX150441
NGEOD625-11|Idaea|COI-5P|JX150444
14
NGEOF067-13|Idaea|COI-5P|KU381399
100
NGEOC1000-11|Idaea|COI-5P|JX149888
NGEOC790-11|Idaea|COI-5P|JX149893
62
NGEOC003-10|Idaea|COI-5P|JF859191
NGEOB757-10|Idaea|COI-5P|JF858961
100
NGEOC338-11|Idaea|COI-5P|JX149908
NGEOB392-10|Idaea|COI-5P|HQ576269
95
NGEOB823-10|Idaea|COI-5P|JF859019
NGEOC276-10|Idaea|COI-5P|JF859449
62
0
NGEOC640-11|Idaea|COI-5P|JX149897
72
GWORD249-07|Idaea AH03Ec|COI-5P
NGEOC122-10|Idaea|COI-5P|JF859304
100
NGEOC064-10|Idaea|COI-5P|JF859249
55
NGEOA250-09|Idaea|COI-5P|GU671931
15
NGEOC050-10|Idaea|COI-5P|JF859236
99
NGEOA228-09|Idaea|COI-5P|GU671947
75
NGEOA303-09|Idaea|COI-5P|GU671894
NGEOA995-09|Idaea|COI-5P|HM425285
64
NGEOC130-10|Idaea|COI-5P|JF859311
NGEOC460-11|Idaea|COI-5P|KU381211
100
NGEOB851-10|Idaea|COI-5P|JF859046
100
0
NGEOD769-11|Idaea|COI-5P|JX150448
10
NGEOF651-13|Idaea|COI-5P|KU381218
NGEOD102-11|Idaea|COI-5P|JX150462

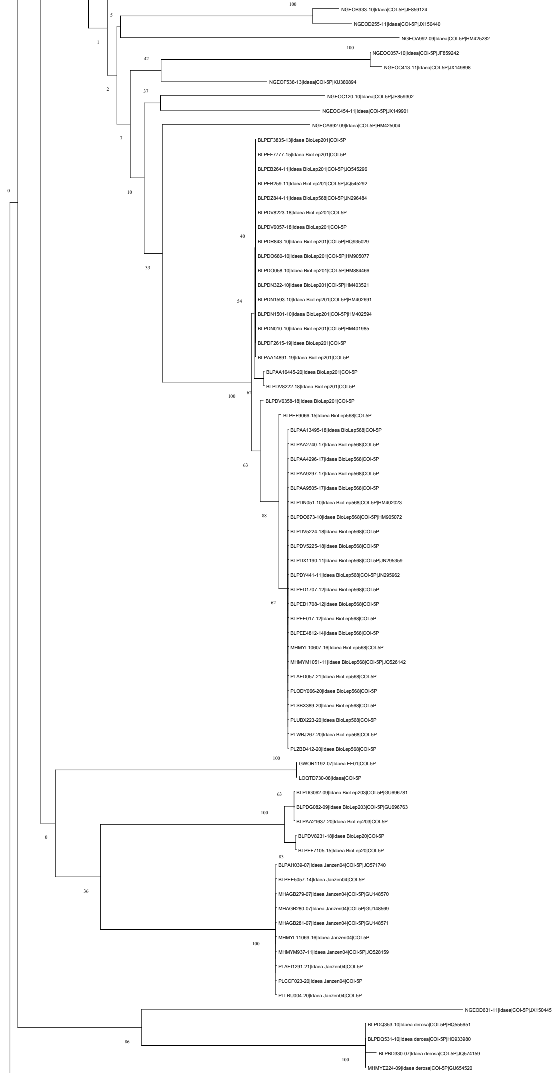
100
NGEOB933-10|Idaea|COI-5P|JF859124
5
NGEOD255-11|Idaea|COI-5P|JX150440
NGEOA992-09|Idaea|COI-5P|HM425282
1
100
NGEOC057-10|Idaea|COI-5P|JF859242
42
NGEOC413-11|Idaea|COI-5P|JX149898
NGEOF538-13|Idaea|COI-5P|KU380894
2
37
NGEOC120-10|Idaea|COI-5P|JF859302
NGEOC454-11|Idaea|COI-5P|JX149901
NGEOA692-09|Idaea|COI-5P|HM425004
7
BLPEF3835-13|Idaea BioLep201|COI-5P
BLPEF7777-15|Idaea BioLep201|COI-5P
BLPEB264-11|Idaea BioLep201|COI-5P|JQ545296
BLPEB259-11|Idaea BioLep201|COI-5P|JQ545292
0
10
BLPDZ844-11|Idaea BioLep568|COI-5P|JN296484
BLPDV8223-18|Idaea BioLep201|COI-5P
BLPDV6057-18|Idaea BioLep201|COI-5P
40
BLPDR843-10|Idaea BioLep201|COI-5P|HQ935029
BLPDO680-10|Idaea BioLep201|COI-5P|HM905077
33
BLPDO058-10|Idaea BioLep201|COI-5P|HM884466
BLPDN322-10|Idaea BioLep201|COI-5P|HM403521
54
BLPDN1593-10|Idaea BioLep201|COI-5P|HM402691
BLPDN1501-10|Idaea BioLep201|COI-5P|HM402594
BLPDN010-10|Idaea BioLep201|COI-5P|HM401985
BLPDF2615-19|Idaea BioLep201|COI-5P
BLPAA14891-19|Idaea BioLep201|COI-5P
BLPAA16445-20|Idaea BioLep201|COI-5P
BLPDV8222-18|Idaea BioLep201|COI-5P
100
62
BLPDV6358-18|Idaea BioLep201|COI-5P
BLPEF9066-15|Idaea BioLep568|COI-5P
BLPAA13495-18|Idaea BioLep568|COI-5P
BLPAA2740-17|Idaea BioLep568|COI-5P
BLPAA4296-17|Idaea BioLep568|COI-5P
63
BLPAA9297-17|Idaea BioLep568|COI-5P
BLPAA9505-17|Idaea BioLep568|COI-5P
BLPDN051-10|Idaea BioLep568|COI-5P|HM402023
88
BLPDO673-10|Idaea BioLep568|COI-5P|HM905072
BLPDV5224-18|Idaea BioLep568|COI-5P
BLPDV5225-18|Idaea BioLep568|COI-5P
BLPDX1190-11|Idaea BioLep568|COI-5P|JN295359
BLPDY441-11|Idaea BioLep568|COI-5P|JN295962
BLPED1707-12|Idaea BioLep568|COI-5P
62
BLPED1708-12|Idaea BioLep568|COI-5P
BLPEE017-12|Idaea BioLep568|COI-5P
BLPEE4812-14|Idaea BioLep568|COI-5P
MHMYL10607-16|Idaea BioLep568|COI-5P
MHMYM1051-11|Idaea BioLep568|COI-5P|JQ526142
PLAED057-21|Idaea BioLep568|COI-5P
PLODY066-20|Idaea BioLep568|COI-5P
PLSBX389-20|Idaea BioLep568|COI-5P
PLUBX223-20|Idaea BioLep568|COI-5P
PLWBJ267-20|Idaea BioLep568|COI-5P
PLZBD412-20|Idaea BioLep568|COI-5P
100
GWOR1192-07|Idaea EF01|COI-5P
LOQTD730-08|Idaea|COI-5P
63
BLPDG062-09|Idaea BioLep203|COI-5P|GU696781
100
BLPDG082-09|Idaea BioLep203|COI-5P|GU696763
BLPAA21637-20|Idaea BioLep203|COI-5P
0
BLPDV8231-18|Idaea BioLep20|COI-5P
BLPEF7105-15|Idaea BioLep20|COI-5P
83
BLPAH039-07|Idaea Janzen04|COI-5P|JQ571740
BLPEE5057-14|Idaea Janzen04|COI-5P
36
MHAGB279-07|Idaea Janzen04|COI-5P|GU148570
MHAGB280-07|Idaea Janzen04|COI-5P|GU148569
MHAGB281-07|Idaea Janzen04|COI-5P|GU148571
MHMYL11069-16|Idaea Janzen04|COI-5P
100
MHMYM937-11|Idaea Janzen04|COI-5P|JQ528159
PLAEI1291-21|Idaea Janzen04|COI-5P
PLCCF023-20|Idaea Janzen04|COI-5P
PLLBU004-20|Idaea Janzen04|COI-5P
NGEOD631-11|Idaea|COI-5P|JX150445
BLPDQ353-10|Idaea derosa|COI-5P|HQ555651
86
BLPDQ531-10|Idaea derosa|COI-5P|HQ933980
BLPBD330-07|Idaea derosa|COI-5P|JQ574159
100
MHMYE224-09|Idaea derosa|COI-5P|GU654520

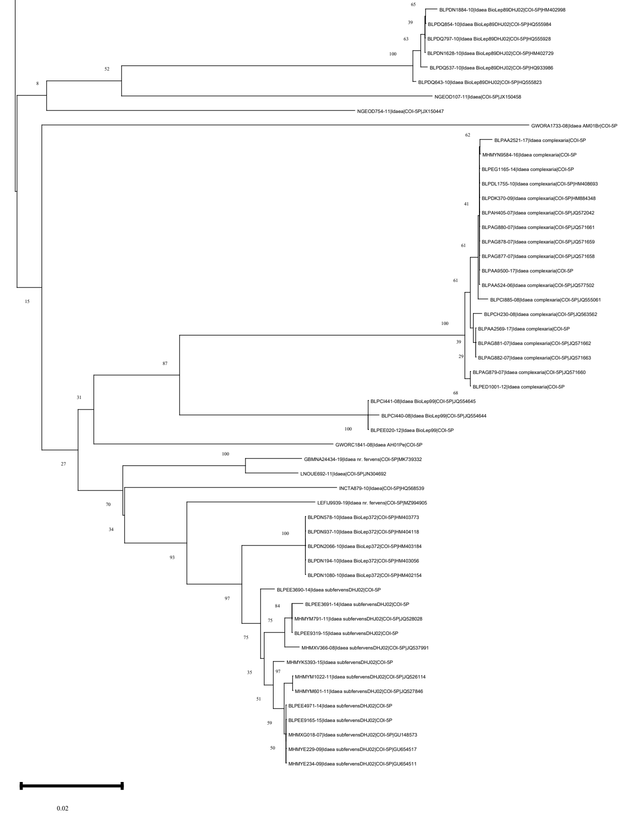
BLPDN1884-10|Idaea BioLep89DHJ02|COI-5P|HM402998
BLPDQ854-10|Idaea BioLep89DHJ02|COI-5P|HQ555984
BLPDQ797-10|Idaea BioLep89DHJ02|COI-5P|HQ555928
BLPDN1628-10|Idaea BioLep89DHJ02|COI-5P|HM402729
BLPDQ537-10|Idaea BioLep89DHJ02|COI-5P|HQ933986
BLPDQ643-10|Idaea BioLep89DHJ02|COI-5P|HQ555823
NGEOD107-11|Idaea|COI-5P|JX150458
NGEOD754-11|Idaea|COI-5P|JX150447
GWORA1733-08|Idaea AM01Br|COI-5P
BLPAA2521-17|Idaea complexaria|COI-5P
MHMYN9584-16|Idaea complexaria|COI-5P
BLPEG1165-14|Idaea complexaria|COI-5P
BLPDL1755-10|Idaea complexaria|COI-5P|HM408693
BLPDK370-09|Idaea complexaria|COI-5P|HM884348
BLPAH405-07|Idaea complexaria|COI-5P|JQ572042
BLPAG880-07|Idaea complexaria|COI-5P|JQ571661
BLPAG878-07|Idaea complexaria|COI-5P|JQ571659
BLPAG877-07|Idaea complexaria|COI-5P|JQ571658
BLPAA9500-17|Idaea complexaria|COI-5P
BLPAA524-06|Idaea complexaria|COI-5P|JQ577502
BLPCI885-08|Idaea complexaria|COI-5P|JQ555061
BLPCH230-08|Idaea complexaria|COI-5P|JQ563562
BLPAA2569-17|Idaea complexaria|COI-5P
BLPAG881-07|Idaea complexaria|COI-5P|JQ571662
BLPAG882-07|Idaea complexaria|COI-5P|JQ571663
BLPAG879-07|Idaea complexaria|COI-5P|JQ571660
BLPED1001-12|Idaea complexaria|COI-5P
BLPCI441-08|Idaea BioLep99|COI-5P|JQ554645
BLPCI440-08|Idaea BioLep99|COI-5P|JQ554644
BLPEE020-12|Idaea BioLep99|COI-5P
GWORC1841-08|Idaea AH01Pe|COI-5P
GBMNA24434-19|Idaea nr. fervens|COI-5P|MK739332
LNOUE692-11|Idaea|COI-5P|JN304692
INCTA879-10|Idaea|COI-5P|HQ568539
LEFIJ9939-19|Idaea nr. fervens|COI-5P|MZ994905
BLPDN578-10|Idaea BioLep372|COI-5P|HM403773
BLPDN937-10|Idaea BioLep372|COI-5P|HM404118
BLPDN2066-10|Idaea BioLep372|COI-5P|HM403184
BLPDN194-10|Idaea BioLep372|COI-5P|HM403056
BLPDN1080-10|Idaea BioLep372|COI-5P|HM402154
BLPEE3690-14|Idaea subfervensDHJ02|COI-5P
BLPEE3691-14|Idaea subfervensDHJ02|COI-5P
MHMYM791-11|Idaea subfervensDHJ02|COI-5P|JQ528028
BLPEE9319-15|Idaea subfervensDHJ02|COI-5P
MHMXV366-08|Idaea subfervensDHJ02|COI-5P|JQ537991
MHMYK5393-15|Idaea subfervensDHJ02|COI-5P
MHMYM1022-11|Idaea subfervensDHJ02|COI-5P|JQ526114
MHMYM601-11|Idaea subfervensDHJ02|COI-5P|JQ527846
BLPEE4971-14|Idaea subfervensDHJ02|COI-5P
BLPEE9165-15|Idaea subfervensDHJ02|COI-5P
MHMXG018-07|Idaea subfervensDHJ02|COI-5P|GU148573
MHMYE229-09|Idaea subfervensDHJ02|COI-5P|GU654517
MHMYE234-09|Idaea subfervensDHJ02|COI-5P|GU654511
0.02
